# Soft Neural Interfaces for Circuit‐Level Analysis of Magnetogenetic Deep Brain Stimulation in Parkinson's Disease Models

**DOI:** 10.1002/adhm.202505548

**Published:** 2026-04-15

**Authors:** Jakyoung Lee, Yeongdo Lee, Enji Kim, Hunkyu Seo, Myoungjae Oh, Hanho Joo, Somin Lee, Jae‐Hyun Lee, Hyun Ho Jung, Minsuk Kwak, Jang‐Ung Park

**Affiliations:** ^1^ Department of Materials Science & Engineering Yonsei University Seoul Republic of Korea; ^2^ Center for Nanomedicine Institute for Basic Science (IBS) Seoul Republic of Korea; ^3^ Department of Nano Biomedical Engineering (NanoBME) Advanced Science Institute Yonsei University Seoul Republic of Korea; ^4^ Department of Neurosurgery Yonsei University College of Medicine Seoul Republic of Korea; ^5^ Yonsei‐KIST Convergence Research Institute Seoul Republic of Korea

**Keywords:** electrophysiological recording, liquid metal, magnetogenetic deep brain stimulation, neural interfaces, soft bioelectronics

## Abstract

Magnetogenetic deep brain stimulation (MG‐DBS) represents a wireless neuromodulation that has demonstrated long‐lasting behavioral benefits in Parkinson's disease models. However, the circuit‐level mechanisms underlying these therapeutic effects have remained uncharacterized due to limitations of conventional neural interfaces. We present a bio‐integrable soft neural interface featuring ultrasoft liquid‐metal probes with bioresorbable stiffeners and customizable interconnects directly printed onto cranial surfaces to match individual skull anatomy and nanoparticle injection sites. This platform enables stable multi‐regional recordings from deep brain structures without chronic tissue damage. We systematically investigate MG‐DBS therapeutic mechanisms in a Parkinson's disease mouse model. Circuit‐level analysis reveals that MG‐DBS modulates pathological beta‐band oscillations and inter‐regional synchrony across the cortico‐basal ganglia‐thalamic circuit. Direct comparison with conventional electrical DBS demonstrates that MG‐DBS effects persisted approximately fifteen‐fold longer after stimulation cessation. Our electrophysiological recordings elucidate the mechanistic basis for this sustained therapeutic effect, providing unprecedented insights into magnetogenetic neuromodulation dynamics.

## Graphical abstract

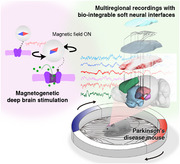

## 1 Introduction

Advances in neural interface technologies have significantly improved the capacity for high‐fidelity monitoring of brain activity, thereby enabling more systematic investigations into the underlying mechanisms of neuromodulation [1, 2, 3]. In parallel, neuromodulation strategies including electrical, optical, and magnetic stimulation have undergone rapid development [4, 5, 6, 7, 8, 9], with these approaches modulating brain activity by inducing depolarization of neuronal membrane potential. Electrical stimulation, delivered through implanted electrodes, can effectively modulate deep brain regions by directly altering membrane potential [10, 11, 12]. However, chronic implantation of rigid electrodes inevitably provokes inflammatory responses and tissue damage, while also posing risks of device failure [13, 14].

Genetic neuromodulation enables cell‐ and circuit‐specific control in vivo with unprecedented precision. Optogenetics uses light to modulate cells that have been genetically engineered to respond to specific wavelengths [15, 16, 17]. Capable of artifact‐free electrophysiology and millisecond temporal precision, it has made remarkable contributions in understanding neural mechanisms of brain functions, but light scattering limits depth and often requires invasive fiber implants [18, 19, 20]. Chemogenetics is tetherless and well‐suited to chronic modulation, yet systemic ligand diffusion yields slow onset and poor temporal precision [21]. Sonogenetics achieves noninvasive, deep, spatially selective activation, but carries risks of off‐target effects and minute‐long after‐responses that constrain precise timing [22]. Recently, magnetogenetics (MG) harnesses the power of magnetic fields for remote and wireless neuromodulation [5, 23, 24, 25, 26]. Magneto‐mechanical‐genetics utilizes nanoscale magneto‐mechanical actuator (m‐Torquer) and a rotating magnetic field generator for mechanically opening Piezo1 ion channels and remote stimulation of targeted deep‐brain neurons in intact, freely moving animals [27, 28, 29]. Unlike conventional neuromodulation strategies, this magneto‐mechanical‐genetics operates without tethered electronics, minimizing chronic invasiveness and allowing wireless, long‐range, large‐area deep brain stimulation (DBS) with cellular and anatomic specificity in nearly any experimental context. This approach has been successfully applied to a Parkinson's disease (PD) mouse model, substantially alleviating the motor dysfunction and yielding sustained therapeutic benefits and relatively long‐lasting behavioral improvements even after cessation of magnetic stimulation [30, 31, 32, 33].

Despite its promise, the circuit‐level mechanisms underlying magnetogenetic deep brain stimulation (MG‐DBS) mitigating PD's motor symptoms remain poorly understood due to the lack of tools for simultaneous neural recording of circuit‐level activity across multiple deep brain regions. Brain function emerges from dynamic interactions between distributed circuits rather than isolated regions, and disruptions in these networks drive neurological disorders like PD [19, 34]. Hence, to uncover how MG‐DBS modulates these interconnected circuits, neural interfaces capable of simultaneous high‐resolution recording across spatially distributed, functionally connected deep brain structures are critically needed.

Conventional surface‐type electrode arrays, widely used in electrocorticography, offer minimally invasive recording capability across broad cortical areas, but their utility is limited to surface‐levels, restricting access to deeper brain structures critical for complex brain functions [35]. Implantable electrodes, also known as neural probes, have been developed to overcome this limitation [36, 37]. However, conventional probes fabricated from rigid silicon or metals cause severe mechanical mismatch with brain tissue, often triggering immune responses [38]. When rigid probes are implanted for extended periods, micromotions arising from respiration and hemodynamics induce scar tissue formation around the probes, which degrades signal quality over time. To address these issues, soft polymer‐based neural probes have been introduced, improving mechanical compliance and reducing tissue damage [39, 40]. Yet, their inherently low buckling force necessitates rigid shuttles or syringes for implantation, reintroducing tissue damage and offsetting the benefits of soft interfaces [41]. Moreover, conventional systems rely on bulky printed circuit boards (PCBs) and rigid interconnections to transmit recorded signals from implanted probes to external devices. These components not only restrict animal mobility but also limit the number of implantable probes due to their cumbersomeness. Consequently, existing technologies face significant challenges in enabling high‐fidelity investigation of circuit‐level brain dynamics across functionally connected brain regions, as they can monitor only a limited number of brain regions and provide access to just a fragment of the neural circuit.

Here, we present a fully bio‐integrable soft neural interface system that was specifically developed to enable stable multi‐regional recordings from deep brain structures during MG‐DBS research in a PD model, thereby facilitating comprehensive observation of circuit‐level neural dynamics. This work provides three major new contributions to the field. First, to elucidate the circuit‐level electrophysiological mechanisms of MG‐DBS, we formed an ultrasoft liquid metal (LM)‐based neural interface with bioresorbable stiffeners and customizable interconnects directly printed onto the cranial surface. Since MG‐DBS research requires nanoparticle (m‐Torquer) injection and neural probe implantation at locations that vary across subjects due to individual skull anatomy, conventional predesigned systems face connectivity challenges. Our platform employed custom‐printed LM interconnections that adapted to each subject's unique anatomy and injection coordinates, ensuring reliable electrical connectivity. The bioresorbable stiffeners enabled reliable implantation without rigid shuttles and dissolved in vivo to minimize tissue trauma. Second, leveraging this bio‐integrable neural interface, we systematically investigated the circuit‐level effects of MG‐DBS in a PD mouse model. MG‐DBS applied to the subthalamic nucleus (STN) alleviated pathological beta‐band oscillations across the cortico–basal ganglia–thalamic (CBT) circuit and selectively suppressed long pathological bursts while preserving physiological ones. These electrophysiological changes were closely aligned with improvements in motor behavior, underscoring the therapeutic relevance of circuit‐level oscillatory control. Third, we directly compared MG‐DBS with conventional electrical DBS (E‐DBS), thereby revealing their differential modulatory mechanisms. Notably, while the therapeutic effects of conventional E‐DBS in a PD model ceased the moment the stimulus was removed, the effects of MG‐DBS persisted for a significantly longer duration, approximately 15 times longer than those of E‐DBS, after the magnetic stimulation was discontinued. Our brain recording experiments were able to pinpoint the electrophysiological basis for this sustained effect, offering a new perspective on the unique modulatory mechanisms of this therapy.

Together, these findings demonstrate that our bio‐integrable soft neural interface provides a powerful platform for dissecting the circuit‐level dynamics of neuromodulation, while positioning MG‐DBS as a promising next‐generation therapy for neurological disorders.

## 2 Results

### 2.1 Magnetogenetic System Preparation and Soft Neural Interface Formation

**FIGURE 1:**
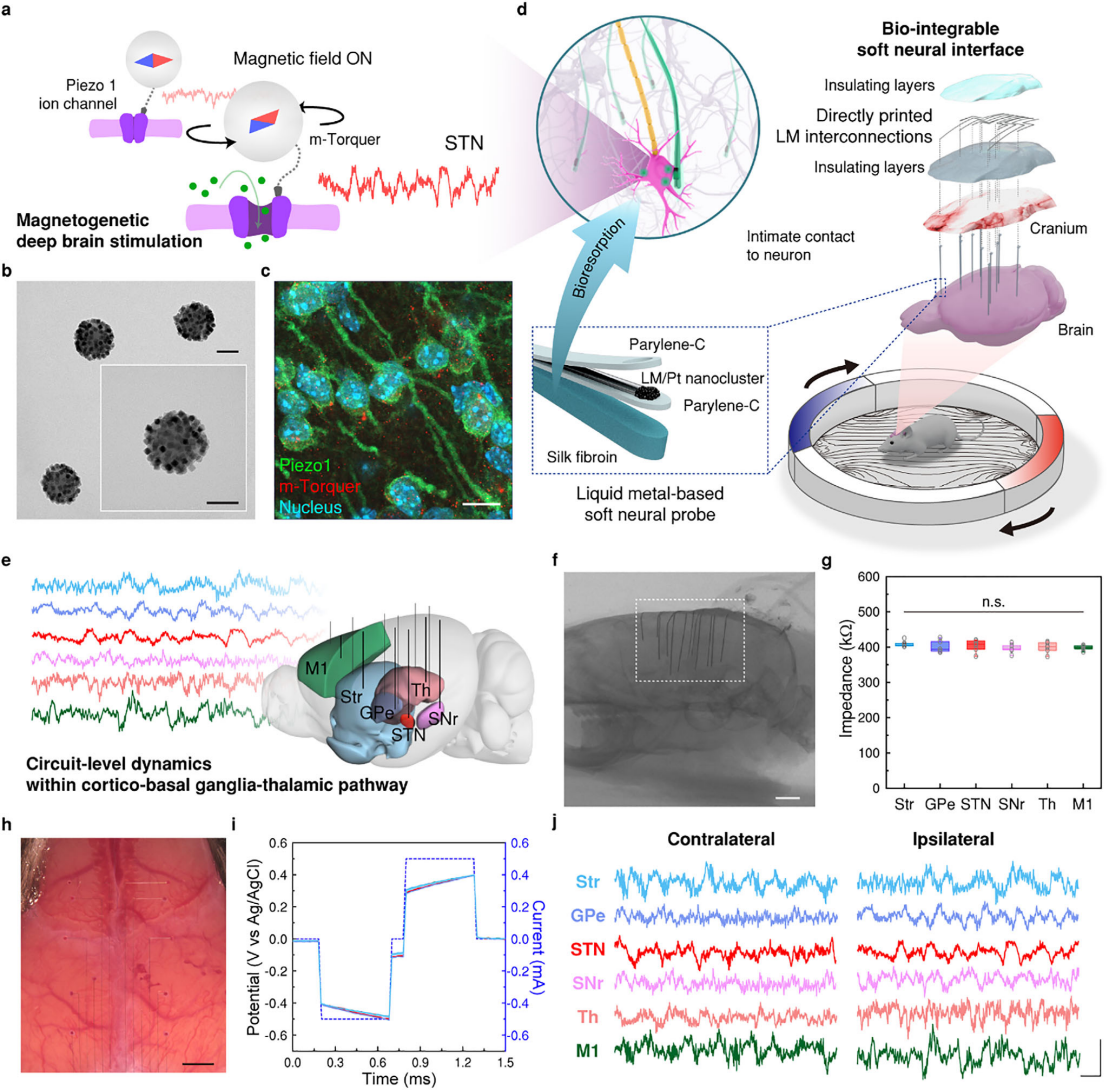
Bio‐integrable soft neural interface and nanoscale magnetic torque actuator. (a) Schematic illustrations describing the mechanism of MG‐DBS in STN. (b) Transmission electron micrograph of m‐Torquer and its magnified image (inset). Scale bars, 100 nm. (c) Immunofluorescence image showing the expression and spatial localization of Piezo1 (green) and m‐Torquer (red) in brain tissue, with colocalization around cell nuclei stained with DAPI (blue). Scale bar, 10 µm. (d) Schematic layouts of soft neural interface integrated with the mouse brain and cranium. The mouse is located inside the magnetic force generator for magnetic stimulation. (e) Schematic illustration describing the monitoring of neural response in the MG‐DBS within the cortico‐basal ganglia‐thalamic (CBT) circuit. Brain image was from Allen Brain Atlas. (f) X‐ray image showing twelve neural probes implanted into bilateral CBT regions. Scale bar, 2 mm. (g) Impedance measurements of LM‐based electrodes implanted across bilateral CBT regions. Individual data points are overlaid (*n* = 3 electrodes per unilateral region; *n* = 6 per each x‐axis point). Box: 25th and 75th percentiles. Line: median. Whiskers: 1.5× interquartile range from the quartiles. (h) Optical stereomicrograph of LM‐based interconnections directly printed onto the cranial surface. Scale bar, 1 mm. (i) Electrical waveforms recorded from twelve individual LM‐based interconnection electrodes (left y‐axis) as a response to biphasic pulse (right y‐axis). (j) Representative local field potential (LFP) recordings obtained from bilateral CBT regions: Str, GPe, STN, SNr, Th, and M1. Scale bars, 500 ms (horizontal), 500 µV (vertical). LM, liquid metal; MG‐DBS, magnetogenetic deep brain stimulation; STN, subthalamic nucleus; CBT, cortico‐basal ganglia‐thalamic; Str, striatum; GPe, globus pallidus externa; SNr, substantia nigra pars reticulata; Th, thalamus; M1, primary motor cortex.

For region‐specific MG‐DBS in PD mice, we stereotaxically delivered a recombinant adenovirus encoding Myc‐tagged Piezo1 (Ad‐Piezo1) into the STN. This enables subsequent binding of m‐Torquer functionalized with anti‐Myc antibodies as illustrated in Figure S1 [27, 30]. Under a rotating magnetic field, the m‐Torquer exerts magnetic torque on Piezo1 channels, inducing calcium influx and neuronal excitation in a cell‐specific manner (Figure 1a). We prepared the viral vector and synthesized m‐Torquer following previously established protocols (Methods; Figure 1b) [27, 28, 30]. Fluorescence histological images demonstrated the successful delivery of both Ad‐Piezo1 and m‐Torquer to the target neuronal population in the STN (Figure 1c, Figure S2). In order to validate the biocompatibility of using m‐Torquer for therapeutic neuromodulation, we injected m‐Torquer into the STN and conducted immunohistochemical analysis after 3 weeks of injection. As comparison groups, we used optic fiber and steel cannula implants, which also deliver stimulation to the STN, analogous to m‐Torquer. Notably, m‐Torquer injection requires only a single insertion of a needle, whereas the optic fiber and steel annulus remained implanted throughout the experimental period to enable neuromodulation. Figure S3a showed that m‐Torquer implantation resulted in a reduced long‐term astrocytic response compared to conventional optic fiber or steel cannula implants, as indicated by GFAP staining, while microglial activation (Iba1) was comparable across groups at the 3‐week post‐implantation time point. These findings suggest improved long‐term biocompatibility of m‐Torquer within brain tissue.

In addition, Figure S3b presents a longitudinal quantification of iron content following m‐Torquer injection. To clarify the temporal stability of the injected material, the analysis was expanded to include 1‐, 2‐, 3‐, and 4‐week time points (weeks 0–4). The results showed no significant change in the relative iron amount over this period, confirming that the injected m‐Torquer remained chemically stable in vivo without measurable loss of iron content during the chronic time window examined.

To investigate circuit‐level neural dynamics during MG‐DBS, we formed a bio‐integrable soft neural interfacing system comprising multiple neural probes and their interconnection lines, all printed using LM, specifically eutectic gallium‐indium alloy (EGaIn; 75.5% gallium, 24.5% indium by weight) (Figure 1d). Due to its exceptionally low elastic modulus (∼200 kPa) [42, 43], LM‐based electrodes significantly reduced the mechanical mismatch with soft brain tissue compared to solid‐state conductive materials (e.g., Silicon: 190 GPa, platinum: 140 GPa, Gold: 70 GPa) [44, 45]. Along with its reliable biocompatibility [46, 47, 48, 49, 50], the high structural deformability of LM further supported stable, long‐term integration of the interface with both dynamic brain tissue and the curved cranial surface. Immunofluorescence staining of STN, one of the implantation regions for electrophysiological recording, at 6 weeks post‐implantation showed that the soft LM probe elicited markedly reduced Iba1‐positive microglial immunoreactivity compared to a rigid tungsten probe (inner diameter, 76.2 µm; A‐M systems), and GFAP immunoreactivity remained comparable to the control tissue (Figure S4).

The LM‐based soft neural probes were designed with neuron‐scale resolution and region‐specific geometry. We utilized our previously reported high‐resolution printing system, which allowed precise control of electrode dimensions by tuning parameters such as nozzle diameter, printing speed, and extrusion pressure (Figure S5a,b) [50, 51]. This technique enabled us to print 5 µm‐width LM electrodes with customizable lengths corresponding to the depth and spatial configuration of the targeted brain regions (Figure S5c). The printed electrodes were encapsulated with a bilayer of parylene‐C film (1 µm thick for the bottom layer and 2.5 µm thick for the top layer), yielding a total probe thickness of 6 µm and a bending stiffness of 0.61 pN·m^2^ (Calculation of bending stiffness is described in Methods). This thin and compliant structure, comparable in scale to mouse brain neurons, facilitated intimate contact with neural tissue and enabled high‐resolution electrophysiological recordings. To further enhance electrical recording fidelity, platinum (Pt) nanoclusters were electroplated onto the LM electrode's open areas for neural contact (58.8 µm^2^), resulting in a significant reduction in electrode impedance compared to the pristine LM electrode, from 995.6 ± 10.70 to 393.2 ± 6.041 kΩ at 1 kHz (Methods; Figures S6 and S7). Notably, after 3 weeks of implantation into mouse brain followed by explantation, the Pt nanocluster coated electrodes maintained a low impedance of 399.4 ± 4.696 kΩ at 1 kHz, supporting the robust in vivo stability of the Pt‐EGaIn interface (Figure S8a). In addition, an accelerated aging test in PBS at 74°C for 8 days (corresponding to ∼6 months under physiological conditions) confirmed no significant impedance degradation (398.1 ± 1.666 kΩ before aging and 400.3 ± 1.631 kΩ after aging; Figure S8b). These results indicate that our LM‐based electrodes can maintain stable neural recording performance over extended periods.

While the soft probes ensure excellent conformability with brain tissue, their low buckling force posed a challenge for deep brain implantation [52, 53]. Previous approaches had utilized tungsten‐based rigid shuttles [37, 54] or syringe‐assisted insertion [55] to deliver soft neural probes into deep brain regions. Although some studies had reported partial recovery of neural function and re‐establishment of probe‐tissue interfaces after implantation using these approaches, they still caused significant damage to local circuits due to their large insertion diameter (>200 µm) [56, 57, 58]. To mitigate this limitation, we used a bioresorbable silk fibroin layer as a transient stiffener. This stiffener provided mechanical support during implantation with a minimal insertion diameter and gradually dissolved within the tissue. Following dissolution, the probe's intrinsically low bending stiffness (∼0.61 pN·m^2^) allowed it to compliantly follow tissue micromotions, enabling stable and long‐term neural interfacing (Figure S9).

To ensure sufficient buckling force (∼1 mN) while minimizing insertion‐induced trauma, optimization of the silk fibroin thickness was necessary. We therefore conducted mechanical modeling, which revealed that a minimum thickness of ∼58.2 µm was required to exceed the 1 mN buckling threshold, considered sufficient for successful penetration into rodent brain tissue (Figure S10 and Note S1) [59]. Based on this result, we optimized the casting volume of a 10 wt.% silk fibroin solution, yielding a film thickness of 60.8 ± 0.17 µm (Figure S11) and a calculated buckling force of 1.15 ± 0.009 mN. Furthermore, to maintain mechanical stiffness of the silk fibroin stiffener during the few minutes of the implantation process and ensure complete resorption thereafter, we modulated the dissolution kinetics through methanol treatment (prior to its insertion into the brain), which promoted β‐sheet formation and delayed degradation [60]. A brief (<5 min) methanol exposure of silk fibroin stiffener provided sufficient mechanical rigidity and enabled complete dissolution within 48 h (Methods; Figure S12). Residual methanol was removed by vacuum drying at 40°C [61, 62], and the absence of measurable cytotoxicity was confirmed using a Live/Dead cell viability assay (Figure S13). After complete drying of methanol, this optimization allowed successful insertion of the soft probes into both 1 wt.% agarose gel and mouse brain without buckling or displacement (Figure S14). The probe fabrication process is provided in Figure S15 and Methods.

### 2.2 Multi‐Regional Neural Probe Implantation and System Integration

To investigate circuit‐level neural dynamics in the magnetogenetically prepared PD mouse model, we stereotaxically implanted our LM‐based soft neural probes into the bilateral hemispheres of six key regions within the CBT circuit, striatum (Str), globus pallidus externa (GPe), STN, substantia nigra pars reticulata (SNr), thalamus (Th), and primary motor cortex (M1), which collectively mediate movement control (Figure 1e). This implantation was performed following surgical exposure and cranium coating with a medical‐grade polymer (Loctite 4011, Henkel, Germany). Detailed procedures for PD modeling and surgical implantation are provided in Methods. Successful targeting was validated by X‐ray imaging (Figure 1f), showing that each probe reached the intended depth without buckling. Additionally, impedance measurements across all implanted sites showed no statistically significant variation, indicating stable and uniform electrode‐tissue interfacing (Figure 1g).

Following probe implantation, LM interconnection lines (width: 8 µm) were directly printed onto the mouse cranium using the high‐resolution printing system (Figure S16). This approach eliminated the need for bulky and rigid PCBs typically required for conventional neural interfaces, as shown in Figure 1h and Figure S17. To prevent signal leakage, the printed lines were then encapsulated with a soft silicone adhesive (Kwik‐sil, World Precision Instruments, USA). Signal fidelity was evaluated by delivering biphasic current pulses to twelve individual channels using a signal generator (3390, Keithley, USA). As shown in Figure 1i and Figure S18, the transmitted waveforms exhibited no detectable distortion or temporal delay, validating the system's capability for crosstalk‐free, simultaneous multi‐region recordings.

With this integrated system combining MG preparation and bio‐integrable soft neural interface, we applied MG‐DBS to the ipsilateral STN and recorded neural dynamics throughout the bilateral CBT circuits. As shown in Figure 1j, local field potentials (LFPs) were successfully recorded from all targeted regions, demonstrating the functional capability of our bio‐integrable soft neural interface for stable, simultaneous multi‐region deep brain recordings in vivo.

### 2.3 MG‐DBS Mediated Modulation of Parkinsonian Pathology

**FIGURE 2:**
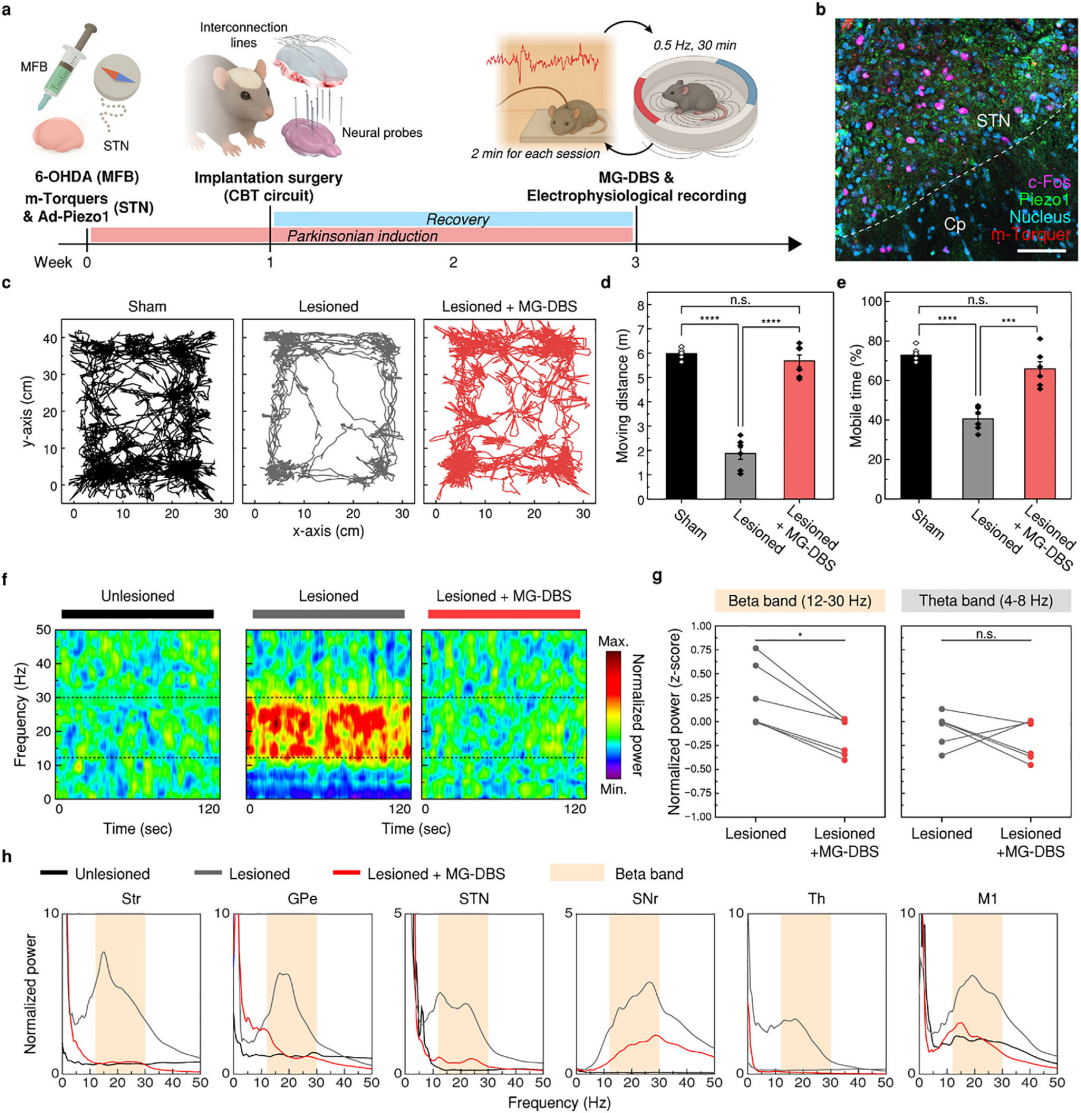
MG‐DBS mediated modulation of Parkinsonian pathology. (a) Schematic timeline showing experimental procedure. (b) Immunofluorescence images of in vivo c‐Fos expression (pink) and m‐Torquer (red) binding in the STN region. Piezo1 (green) and nuclei (DAPI, blue) are also colocalized. Scale bar, 50 µm. (c) Representative trajectory map tracking movement of sham (black), lesioned (grey), and MG‐DBS treated lesioned (lesioned + MG‐DBS) (red) mouse. (d,e) Locomotor activity in sham (black), lesioned (grey), and lesioned + MG‐DBS (red) mice during the test period. Total moving distance (*p* = 1.06 × 10^−6^ for sham and lesioned; *p* = 5.61 × 10^−4^ for lesioned and lesioned + MG‐DBS) (d), and mobile time (*p* = 5.61 × 10^−4^ for sham and lesioned; *p* = 1.20 × 10^−6^ for lesioned and lesioned + MG‐DBS) (e). Data represented mean ± s.e.m. Individual data points are overlaid (*n* = 6 mice per group). Statistical differences were determined with unpaired t‐test; ***p < 0.001, ****p < 0.0001. (f) Representative time‐frequency spectrograms of normalized power in STN for unlesioned (grey), lesioned (blue), and lesioned + MG‐DBS (red) groups. (g) Changes in STN normalized power changes in beta band(12–30 Hz; left, *p* = 0.011) and theta band (4–8 Hz; right) induced by MG‐DBS. All data points are indicated in the graphs (*n* = 6 mice). Statistical differences were determined with a paired *t*‐test; *p < 0.05. (h) Power spectral analysis across the CBT regions for unlesioned (grey), lesioned (blue), and lesioned + MG‐DBS (red) group. Orange shaded zone indicates beta frequency range. MFB, medial forebrain bundle; 6‐OHDA, 6‐hydroxydopamine; Cp, cerebral peduncle.

Figure 2a illustrates the experimental timeline for investigating MG‐DBS‐induced circuit‐level electrophysiological dynamics in a PD mouse model using our bio‐integrable soft neural interface. To establish the PD mouse model, we unilaterally injected 6‐hydroxydopamine (6‐OHDA) into the medial forebrain bundle (MFB), which induced selective degeneration of dopaminergic neurons (Methods) [63, 64]. We confirmed successful lesioning through immunohistochemical analysis, which revealed a significant reduction in tyrosine hydroxylase (TH) expression, a specific marker for dopaminergic neurons, in both the substantia nigra (SN) and Str (Figure S19). During the same surgical procedure, we co‐delivered Ad‐Piezo1 and m‐Torquer to the ipsilateral STN to enable target‐specific MG‐DBS. Subsequent histological analysis demonstrated uniform m‐Torquer distribution throughout the STN volume (Figure 2b, Figure S20). Furthermore, robust c‐Fos expression was observed exclusively under magnetic stimulation, in the targeted hemisphere, while neither m‐Torquer accumulation nor c‐Fos activation was detected in the contralateral hemisphere, confirming the spatial specificity of our approach.

One week following 6‐OHDA lesioning and m‐Torquer delivery, we bilaterally implanted soft neural probes into six distinct regions of the CBT circuit, totaling twelve recording sites. We then conformally printed LM interconnects onto the cranial surface, establishing a stable neural interface capable of monitoring pathological neural dynamics in the PD mouse model. The recording sessions commenced two weeks post‐implantation, a timepoint that coincides with both peak Ad‐Piezo1 expression [28] and the establishment of robust PD pathology. This timing also ensured complete dissolution of the silk fibroin stiffener and adequate post‐surgical recovery, thereby facilitating intimate probe‐neuron contact and reliable neural signal acquisition.

For MG‐DBS administration, we employed a custom‐designed 70 cm‐diameter arena equipped with ten permanent magnets that generated a uniform rotating magnetic field (|B| = 52.6 mT at 0.5 Hz), following our previously reported protocol (Methods; Figure S21) [30]. The stimulation sessions lasted 30 min. This duration was selected based on a duration‐dependent behavioral test (Figure S22). To ensure high‐quality electrophysiological recordings, we performed all recordings under anesthesia with the animal's head secured in a stereotaxic frame to minimize motion artifacts. Each recording session, conducted within a Faraday cage to eliminate external electromagnetic interference, lasted 2 min. We processed all recorded signals using Matlab (version R2023b; Mathworks) (Methods; Figure S23).

Behavioral assessment revealed that 6‐OHDA‐lesioned mice exhibited pronounced motor deficits compared to the sham controls (saline‐injected MFB). MG‐DBS treatment significantly ameliorated these motor impairments, restoring locomotor parameters, including total distance traveled and mobile time, to levels statistically indistinguishable from those of sham animals (Figure 2c–e). These results demonstrate the therapeutic efficacy of MG‐DBS in reversing PD‐associated motor dysfunction.

To elucidate the electrophysiological mechanisms underlying the observed behavioral improvements, we analyzed neural oscillatory activity with particular emphasis on the beta frequency band (12–30 Hz), a well‐established pathological biomarker of PD [65, 66, 67, 68, 69]. Time‐frequency spectrograms from the STN, the direct target of MG‐DBS, revealed that markedly elevated beta‐band power in the lesioned hemisphere compared to the intact contralateral side (Figure 2f, left and center panels). Critically, MG‐DBS treatment substantially suppressed this pathological beta activity (Figure 2f, center and right panels). Quantitative analysis confirmed a statistically significant reduction in normalized beta power following stimulation (*p* = 0.0109), whereas other frequency bands, including the theta band (4‐8 Hz), showed no significant changes (Figure 2g). This frequency‐specific modulation suggests that the effect reflects targeted therapeutic action rather than non‐specific neural suppression.

To determine whether the therapeutic effects of MG‐DBS extend beyond the local stimulation site, we analyzed recordings from the remaining five CBT regions. Time‐frequency spectrograms consistently demonstrated MG‐DBS‐induced suppression of beta‐band activity across all recorded regions (Figure S24). Power spectral analysis revealed that the pathologically elevated beta oscillations observed throughout the ipsilateral CBT regions (Figure 2h, grey traces) were significantly attenuated following MG‐DBS (Figure 2h, red traces), approaching levels comparable to those in the healthy contralateral hemisphere (Figure 2h, black traces). A residual beta peak, particularly prominent in M1, likely reflects the anesthetized resting state of the animal during recordings [70].

Throughout the deployment of our bio‐integrable soft neural interface, which enables simultaneous monitoring of pathological neural dynamics across multiple brain regions, we demonstrated that STN‐targeted MG‐DBS exerts modulatory effects that extend far beyond the local stimulation site to encompass the entire CBT circuit. This circuit‐wide modulation of pathological oscillations, coupled with the observed behavioral improvements, underscores the potential of MG‐DBS as a network‐level therapeutic strategy for PD. The ability to non‐invasively modulate distributed neural circuits through targeted MG stimulations represents a significant advance in neuromodulation technology with promising clinical implications.

### 2.4 Inter‐Regional Synchrony Modulation Within the CBT Circuit by MG‐DBS

**FIGURE 3:**
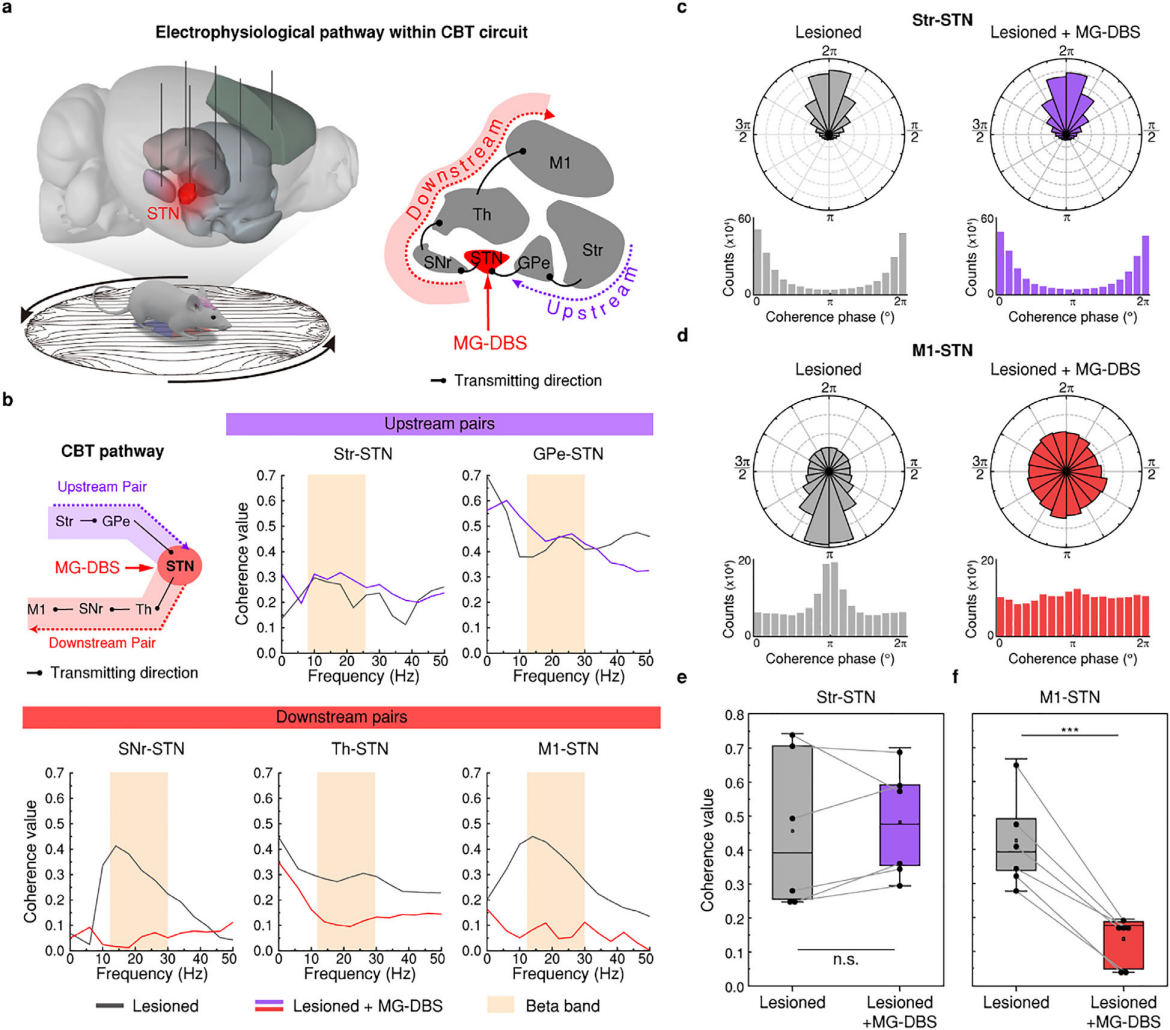
Direction‐dependent modulation of inter‐regional synchrony within the CBT circuit. (a) Schematic illustrations showing CBT circuit in mouse brain, where multiple neural probes are implanted (left) and transmitting direction within the circuit (right). STN is a target region of MG‐DBS (red). Remaining regions are categorized relative to the STN: upstream (Str, GPe) and downstream (SNr, Th, M1) regions. b, Representative coherence values according to frequency range (0 – 50 Hz) in five pairs with STN. Inset illustration exhibited transmitting direction of electrophysiological pathway of CBT circuit. (c,d) Representative histogram of coherent phase for beta band oscillatory synchrony between region pairs: upstream Str‐STN (lesioned, *r* = 0.5686, *θ* = 2.153°; lesioned + MG‐DBS, *r* = 0.5519, *θ* = 2.079°) (c) and downstream M1‐STN (lesioned, *r* = 0.2515, *θ* = 181.8°; lesioned + MG‐DBS, *r* = 0.0400, *θ* = 210.0°) (d) for lesioned (grey) and lesioned + MG‐DBS (purple, red) mouse. (e,f) Quantification of coherence values for each pair: no significant change in upstream Str‐STN coherence (e); significant reduction in M1‐STN (*p* = 0.000964) coherence (f). Individual data points are overlaid (*n* = 6 mice). Box: 25th and 75th percentiles. Line: median. Whiskers: 1.5× interquartile range from the quartiles. Statistical differences were determined with a paired t‐test; ***p < 0.001.

Neural circuits orchestrate brain function through dynamic coordination of electrophysiological activity across interconnected regions. The CBT circuit, which governs voluntary motor control, operates through directional signal transmission from the basal ganglia through the Th to the M1, where motor commands are ultimately executed. In PD, dopaminergic neuron degeneration disrupts this finely tuned coordination, resulting in pathologically synchronized neural activity. Recent evidence indicates that the CBT circuit can resonate with beta‐frequency inputs, producing amplified and phase‐locked oscillations at the network‐level [71, 72]. This observation suggests that pathological coupling in PD may emerge not solely from local dysfunction within individual brain regions, but rather from aberrant synchronization across distributed nodes of the motor circuit. To investigate whether MG‐DBS modulates this pathological inter‐regional synchrony, we employed our soft neural interface system to perform simultaneous recordings from six anatomically defined regions within the CBT network (Figure 3a). We specifically analyzed phase coherence between the stimulated STN and five interconnected regions to quantify MG‐DBS‐induced changes in functional connectivity across the circuit.

We computed coherence spectra to quantify frequency‐resolved phase coupling between the STN and five other regions, which we categorized as either upstream (Str, GPe) or downstream (SNr, Th, M1) based on their anatomical position relative to signal flow through the STN (Figure 3b). Coherence values, calculated from the consistency of phase differences between oscillatory signals from each region pair, served as a measure of functional connectivity strength. In the lesioned state, we observed elevated coherence within the beta frequency range (12–30 Hz) across all region pairs (Figure 3b, grey traces), confirming the presence of pathological hypersynchrony characteristic of PD.

Remarkably, MG‐DBS exhibited direction‐dependent effects on this pathological synchrony (Figure 3b, purple and red traces). In downstream pairs (SNr‐STN, Th‐STN, M1‐STN), MG‐DBS robustly suppressed beta‐band coherence. In contrast, coherence in upstream pairs (Str‐STN, GPe‐STN) remained largely unaffected within the beta band, suggesting selective modulation of descending pathways. Phase distribution analysis further elucidated these directional effects (Figure 3c,d, Figure S25a–c). In the lesioned state, all STN pairs exhibited highly concentrated, unimodal phase distributions, indicative of pathological phase‐locking. Following MG‐DBS, downstream pairs showed disrupted phase‐locking with more uniform phase distributions, while upstream regions maintained their unimodal alignment with only a minor phase shift. Quantitative analysis across six PD mice confirmed this directional specificity (Figure 3e,f, Figure S25d–f). MG‐DBS significantly reduced coherence values in downstream pairs (SNr‐STN, *p* = 0.000071; Th‐STN, *p* = 0.002; M1‐STN, *p* = 0.000964), while producing no statistically significant changes in upstream connectivity. These findings demonstrate that MG‐DBS selectively disrupts pathological synchronization in the descending output pathways of the CBT circuit while preserving afferent signal processing toward the STN.

### 2.5 Beta Burst Shortening by MG‐DBS

Beyond tonic inter‐regional synchrony, beta burst activity represents another critical hallmark of pathological coordination in PD circuits. Beta bursts are transient, high‐amplitude oscillatory events arising from synchronized activity within local neuronal populations [69]. Unlike sustained beta power elevation, which reflects continuous oscillatory activity, these discrete burst events are increasingly recognized as specific pathological signatures that correlate more strongly with motor impairment in PD. Recent evidence indicates that long‐duration beta bursts (>300 ms) are particularly associated with Parkinsonian motor symptoms, whereas shorter bursts represent normal physiological processes with limited pathological relevance [73, 74]. Clinical studies further support this distinction, demonstrating that dopaminergic medication reduces pathological beta activity primarily by shortening burst duration rather than eliminating bursts [75, 76]. Based on these insights, we utilized our soft neural interface to characterize the spatiotemporal dynamics of beta bursts and evaluate how MG‐DBS modulates these transient events across the CBT circuit.

**FIGURE 4:**
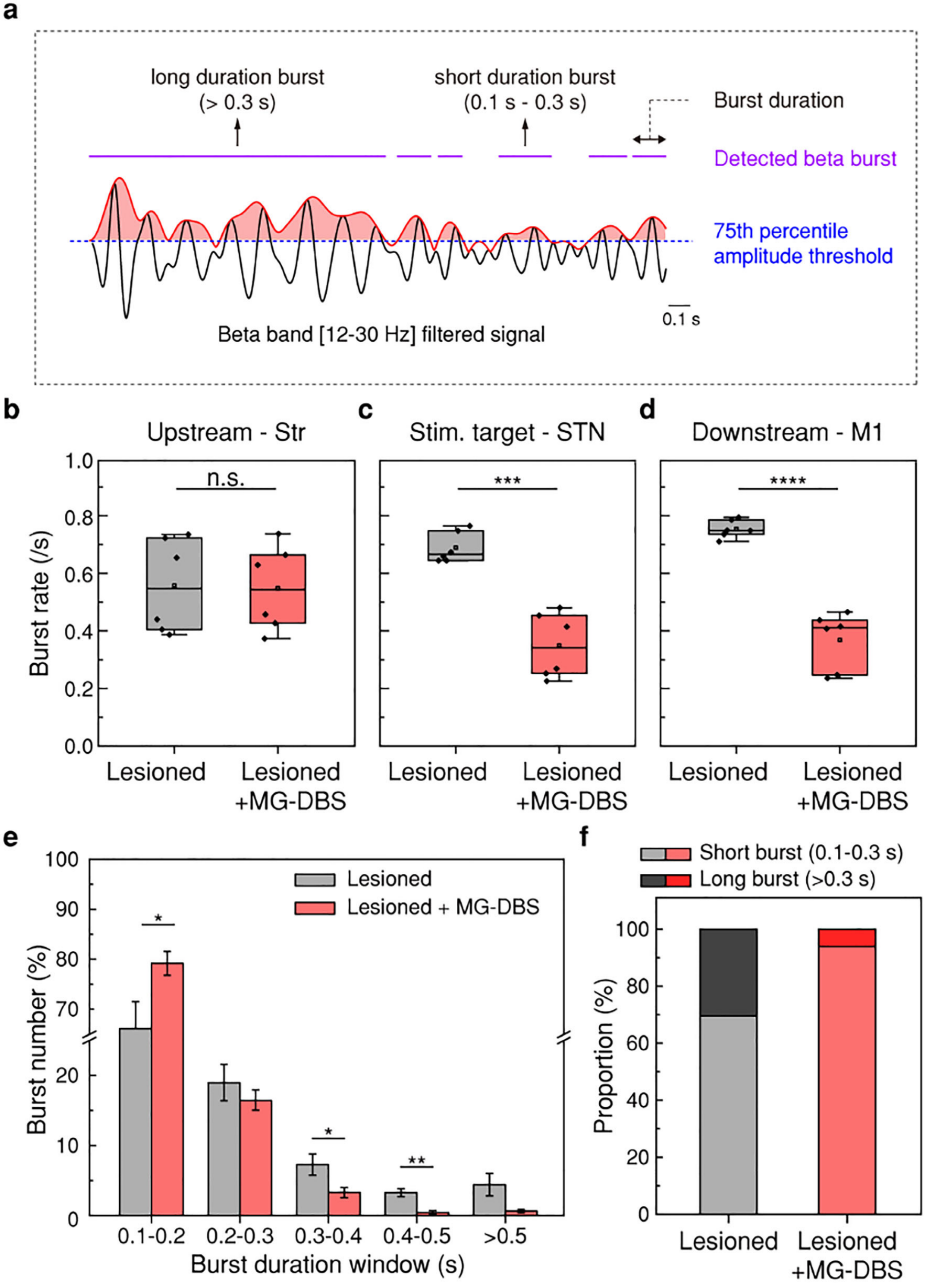
Modulation of beta burst activity by shortening burst duration. (a) Schematic of beta burst detection from a beta frequency range bandpass‐filtered signal (black line) using the 75th percentile amplitude threshold (blue dotted line). Segments exceeding the threshold are defined as beta bursts (purple horizontal line). (b–d) Burst rate changes by MG‐DBS in upstream (Str) (b), stimulation target (STN; *p* = 0.00038) (c), and downstream (M1, *p* = 8.00 × 10^−5^) (d) regions. Individual data points are overlaid (*n* = 6 mice for each group). Box: 25th and 75th percentiles. Line: median. Whiskers: 1.5x interquartile range from the quartiles. Statistical differences were determined with paired *t*‐test; ***p < 0.001, ****p < 0.0001. (e) Distribution of beta bursts across different duration windows for lesioned (grey, *n* = burst events during recording period) and lesioned + MG‐DBS (red, *n* = burst events during recording period) group. Statistical differences were determined with paired *t*‐test; *p < 0.05, **p < 0.01. (f) Representative distribution of short (0.1–0.3 s) and long (>0.3 s) beta bursts. The proportion of short and long bursts is shown for the lesioned state and following MG‐DBS treatment.

Figure 4a illustrates our burst detection methodology. We applied bandpass filtering (12–30 Hz) to LFP signals (black trace) and computed the amplitude envelope (red trace). Bursts were identified as segments where the envelope exceeds the 75th percentile threshold (blue dotted line) with suprathreshold segments highlighted in red. We classified detected bursts by duration (purple horizontal lines): short bursts (100–300 ms) and long bursts (>300 ms), excluding events shorter than 100 ms to minimize noise contributions [77]. These burst detection criteria were adopted in accordance with previously established definitions in Parkinson's disease studies to ensure objective analysis [75, 76].

Analysis of burst rates across the CBT circuit revealed spatially selective modulation by MG‐DBS. In upstream regions (Str, GPe), MG‐DBS did not significantly alter burst rates (Figure 4b, Figure S26a). Conversely, we observed marked reductions in the stimulated STN and all downstream regions, including SNr, Th, and M1 (STN: *p* = 0.00038, SNr: *p* = 0.0001, Th: *p* = 0.00015, M1: *p* = 0.00008) (Figure 4c,d, Figure S26b,c). This spatial pattern mirrors the direction‐selective effects observed in phase coherence analyses (Figure 3), further supporting the hypothesis that MG‐DBS selectively suppresses pathological synchronization along descending pathways. Notably, while beta power showed relatively uniform suppression across all CBT regions (Figure 2h), this spatially selective burst rate reduction suggests that burst dynamics may be more mechanistically linked to the therapeutic effects of MG‐DBS.

To characterize burst modulation in detail, we analyzed individual beta events detected in the STN by plotting amplitude versus duration relationships (Figure S27). While the positive correlation between amplitude and duration persisted, the overall distribution shifted toward shorter durations and lower amplitudes following MG‐DBS. Quantitative analysis using 100 ms duration bins revealed the specificity of this modulation (Figure 4e). MG‐DBS significantly reduced long‐duration bursts in the 0.3–0.4 s and 0.4–0.5 s windows (*p* = 0.0432 and *p* = 0.0024, respectively), while simultaneously increasing short bursts in the 0.1–0.2 s window (*p* = 0.0234). The resulting shift in burst duration distribution (Figure 4f, Figure S28) demonstrates selective truncation of pathological long bursts. Given that long‐duration beta bursts correlate with motor impairment while short bursts reflect normal physiological activity, our findings suggest that MG‐DBS alleviates pathological synchrony not through indiscriminate power reduction, but by specifically reshaping burst dynamics toward a more physiological profile.

### 2.6 The Sustained Neuromodulatory Effect of MG‐DBS

**FIGURE 5:**
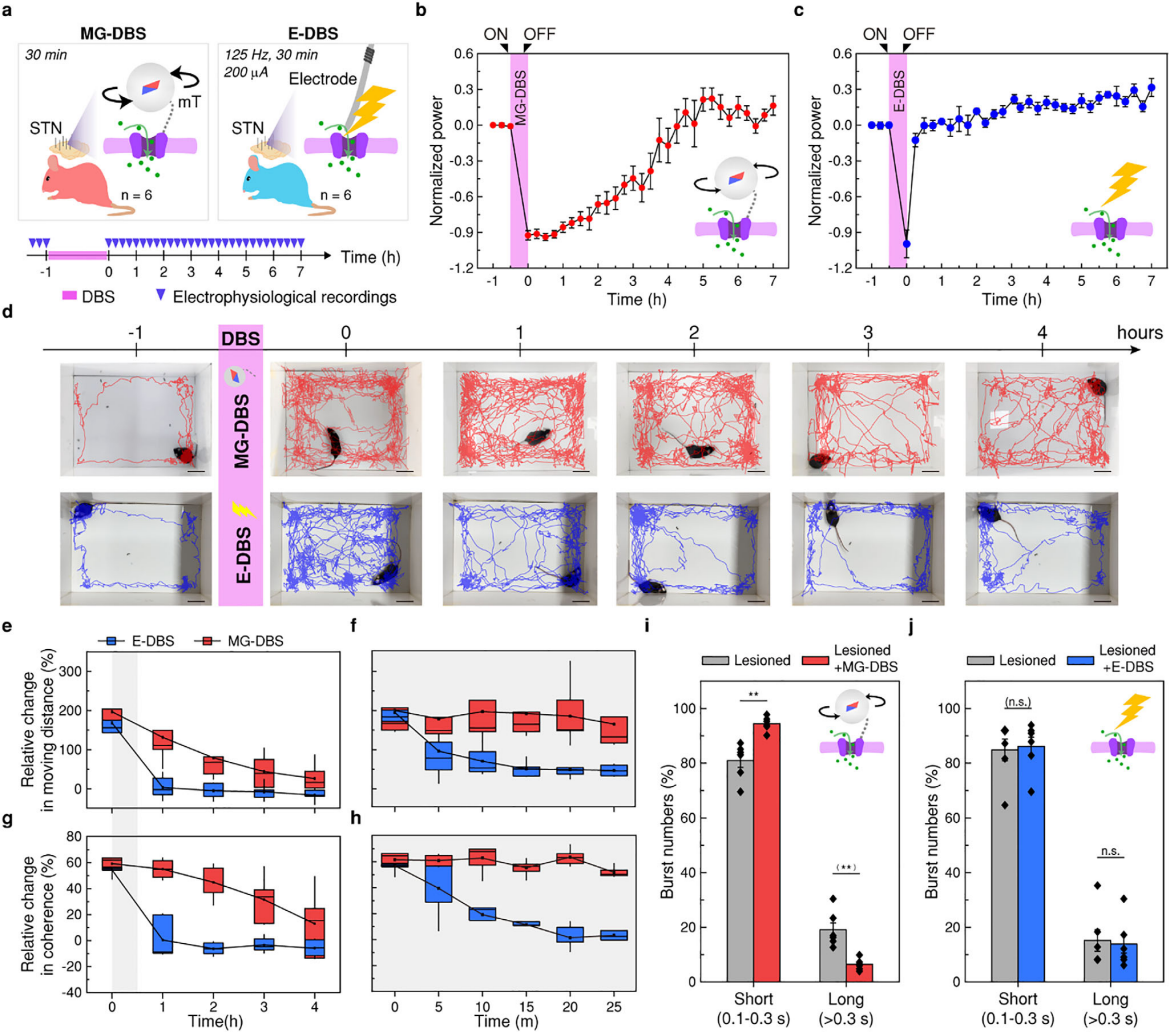
Differential effects of MG‐DBS and E‐DBS on beta power, coherence, and beta burst characteristics. (a) Schematic illustrations of MG‐DBS (left) and conventional electrical DBS (E‐DBS, right) applied to the STN in PD mice. (b,c) Time‐dependent changes in normalized beta band power recorded in the STN following MG‐DBS (b) and E‐DBS (c). Data represented mean ± s.e.m. (d) Representative locomotor trajectories of lesioned mice with MG‐DBS (top, red) and E‐DBS (bottom, blue). (e,f) Quantification of relative changes in moving distance (e) and locomotion in 5‐minute intervals (f) normalized to the pre‐stimulation baseline. (g,h) Relative changes in STN‐M1 coherence following MG‐DBS (red) and E‐DBS (blue) shown as overall changes (g) and detailed time‐resolved changes in 5‐minute intervals (f). (i,j) Quantification of beta bursts distributions in the STN following MG‐DBS (i) and E‐BS (j), categorized into short (0.1–0.3 s) and long (>0.3 s) durations. Data represented mean ± s.e.m. Individual data points are overlaid (*n* = 6 mice for each group). Box: 25th and 75th percentiles. Line: median. Whiskers: 1.5× interquartile range from the quartiles. Statistical differences were determined with a paired *t*‐test; **p < 0.01. 6‐OHDA, 6‐hydroxydopamine; mT, m‐Torquer.

Previous work demonstrated that MG‐DBS induces prolonged motor improvement in PD models [30]. To characterize the electrophysiological basis of this sustained effect and evaluate its therapeutic advantages, we systematically compared post‐stimulation neural dynamics between MG‐DBS and conventional E‐DBS using our soft neural interface. We divided twelve 6‐OHDA‐induced PD mice into two treatment groups: six received MG‐DBS, and six underwent E‐DBS using Pt‐Ir electrodes (inner diameter, 50.8 µm; A‐M systems) (Figure 5a). E‐DBS parameters were set as 125 Hz and 200 µA, following established protocols (Methods) [78]. Following recovery from probe implantation, both groups received 30 min of STN stimulation. We recorded electrophysiological signals at baseline (Time = −1 h), immediately post‐stimulation (Time = 0 h), and at 15‐minute intervals thereafter to track temporal dynamics. During each recording session, electrophysiological signals were acquired for 2 min from awake mice (Figure S29).

Figure 5b,c shows the temporal evolution of normalized beta band power in the STN following MG‐DBS and E‐DBS, respectively. Both modalities induced immediate beta power reduction; however, the duration of suppression differed dramatically. MG‐DBS produced statistically significant beta suppression lasting 3.5 h post‐stimulation, followed by gradual recovery to baseline (Figure 5b). In stark contrast, E‐DBS resulted in a rapid beta rebound, with power returning to baseline within 15 min of stimulation cessation (Figure 5c).

Behavioral assessment corroborated these electrophysiological findings. Open‐field tests conducted hourly (5‐minute tracking session) revealed that MG‐DBS‐treated mice maintained locomotor improvements that gradually diminished over 4 h. Conversely, E‐DBS mice rapidly reverted to pre‐stimulation motor impairment immediately following stimulation offset (Figure 5d, Movie S1). Quantitative analysis of relative changes in moving distance further highlighted these differences (Figure 5e). At 0 h, both groups showed increased locomotion. However, while E‐DBS effects diminished to near‐baseline within 1 h, MG‐DBS mice maintained elevated locomotion beyond 1 h before gradual decline. To capture fine‐scale dynamics during the critical early rebound phase (0–1 h), we performed continuous 30‐minute open‐field tracking with 5‐minute bin analysis (Figure 5f). MG‐DBS preserved therapeutic benefits throughout this period, whereas E‐DBS effects dissipated within approximately 10 min, underscoring the marked difference in effect duration.

We extended our analysis to circuit‐level dynamics by examining temporal changes in M1‐STN beta coherence. Consistent with behavioral outcomes, MG‐DBS induced sustained coherence reduction that gradually recovered over 4 h (Figure 5g). E‐DBS produced only transient suppression, with coherence rebounding within 1 h. High‐resolution analysis at 5‐minute intervals during the initial 30 min confirmed these distinct temporal profiles (Figure 5h). Remarkably, MG‐DBS sustained both electrophysiological and behavioral effects approximately 15‐fold longer than E‐DBS.

Finally, we assessed beta burst dynamics in the STN, classifying events as short (0.1–0.3 s) or long (>0.3 s) duration. MG‐DBS selectively reduced long‐duration bursts while increasing short‐duration bursts, indicating a shift toward a physiological burst pattern (Figure 5i). In contrast, while E‐DBS reduced the overall burst occurrence (Figure S30), it showed no duration‐specific selectivity (Figure 5j).

Previous studies demonstrated that adaptive E‐DBS (aDBS), which delivers stimulation contingent on pathological activity detection, selectively shortens long‐duration beta bursts, a specificity linked to superior therapeutic efficacy compared to conventional E‐DBS [75, 76]. Our results reveal that MG‐DBS exhibited burst modulation patterns remarkably similar to aDBS, selectively truncating pathological long bursts while preserving physiological short bursts. In contrast, conventional E‐DBS indiscriminately suppressed beta activity without duration selectivity. Critically, only MG‐DBS produced sustained improvements in both neural dynamics and motor behavior. These observations suggest that duration‐selective burst modulation may represent a key mechanism underlying the prolonged therapeutic effects of MG‐DBS. Collectively, these findings demonstrate that our soft, bio‐integrable neural interface enables stable, multi‐regional recordings from deep brain structures, facilitating the identification of circuit‐level electrophysiological mechanisms that distinguish different neuromodulation strategies.

## 3 Conclusion

MG‐DBS offers a promising, non‐invasive strategy for subcortical neuromodulation. However, the absence of a suitable neural interface capable of stable, multi‐regional recordings has limited direct investigation into how MG‐DBS influences brain connectivity and circuit‐level activity. To address this gap, we developed a fully bio‐integrable LM‐based soft neural interface. Our approach takes advantage of a distinctive property of MG‐DBS: its therapeutic effects persist beyond the stimulation period. This feature enables electrophysiological analysis before and after stimulation using ultrasoft, neuron‐scale probes capable of simultaneous multi‐regional recording. The excellent compliance of LM ensures stable coupling with brain tissue, effectively overcoming the mechanical mismatch inherent in conventional solid electrodes. To achieve precise targeting of deep brain regions, we incorporated a bioresorbable stiffener that allows accurate implantation of these ultrasoft probes without relying on traumatic rigid shuttles. This design resolves the long‐standing paradox between the low modulus required for long‐term biocompatibility and the stiffness needed for reliable insertion. Furthermore, LM interconnects, custom‐printed to conform to individual skull anatomy and nanoparticle injection coordinates, provide robust and stable connectivity across multiple probes. By uniting these elements, our platform enables direct, multi‐regional electrophysiological analysis of MG‐DBS, capturing circuit‐level dynamics.

Our multi‐regional recording capability revealed previously inaccessible insights into how MG‐DBS reshapes pathological neural dynamics across the CBT circuit. The discovery of direction‐selective modulation, wherein MG‐DBS specifically altered burst dynamics and phase coherence in regions downstream of the STN while preserving upstream activity, challenges the conventional view that DBS operates through sophisticated network‐level mechanisms. The intervention selectively disrupts pathological signal propagation along the SNr‐Th‐M1 axis while maintaining afferent information flow, which may explain the good therapeutic profile observed with MG‐DBS. The selective truncation of long‐duration beta bursts represents another mechanistic advance in understanding neuromodulation efficacy. Previous studies established that pathological long bursts (>300 ms) correlate strongly with motor impairment in PD. Our findings reveal that MG‐DBS spontaneously exhibits the therapeutically optimal modulation pattern. The technique suppresses pathological long bursts while preserving physiological short bursts, without requiring complex closed‐loop control systems. This intrinsic selectivity may arise from the unique activation kinetics of mechanosensitive ion channels, which differ fundamentally from the instantaneous electrical depolarization induced by conventional electrical stimulation.

Our data demonstrate a ∼15‐fold prolongation of therapeutic effects with MG‐DBS compared to conventional electrical DBS, with persistence of beta suppression, coherence reduction, and motor improvement. This sustained modulation suggests that MG‐DBS induces plastic changes in circuit dynamics rather than merely providing temporary symptomatic relief. The underlying mechanism likely involves unique cellular responses to mechanotransduction, as mechanical activation of Piezo1 channels leads to sustained calcium influx and initiates intracellular signaling cascades—such as MAPK and CREB pathways—that promote long‐term synaptic plasticity and neuronal excitability modulation beyond the stimulation period [79, 80, 81, 82, 83].

Translation of these findings into clinical applications will require comprehensive long‐term studies in PD patients to establish safety profiles, optimize stimulation parameters, and validate therapeutic efficacy across diverse patient populations. Such clinical trials would necessitate extensive regulatory approval processes and longitudinal follow‐up studies with extended observation periods to assess both immediate therapeutic benefits and potential long‐term neuroplastic changes

This work establishes a technological framework for investigating wireless neuromodulation mechanisms at the circuit level. The combination of non‐invasive stimulation, sustained effects, and burst‐selective modulation positions MG‐DBS as a potentially transformative treatment for movement disorders. The wireless nature eliminates infection risks while enabling intermittent treatment schedules. By providing the first comprehensive characterization of circuit‐level mechanisms underlying MG neuromodulation, these findings advance our understanding of how external magnetic fields can therapeutically reshape pathological brain dynamics and highlight the potential for wireless, minimally invasive approaches to achieve superior clinical outcomes in neurological disorders.

## 4 Experimental Section/Methods

### 4.1 Fabrication of Neural Probes

#### 4.1.1 Preparation of Bioresorbable Temporal Stiffener

A 1.5 cm × 1.5 cm silicon wafer was thoroughly cleaned and treated with ultraviolet ozone (UVO) for 15 min to enhance surface wettability. An adhesion layer of Surpass was spin‐coated at 4000 rpm for 40 s. Subsequently, 5 w/v% cellulose acetate (in acetone) was spin‐coated at 3000 rpm for 30 s to enable facile release of the probes after fabrication. The substrate was then baked at 60°C for 5 min to stabilize the film.

To form the temporary stiffener, 180 µl (80 µL/cm^2^) of 10 wt.% silk fibroin solution was drop‐cast onto the cellulose acetate‐coated wafer at room temperature. To prolong the dissolution time of the silk fibroin and maintain mechanical rigidity during implantation, the silk fibroin film was shortly immersed (<5 min) in 70% methanol at room temperature to induce β‐sheet formation. After treatment, the film was vacuum‐dried at 40°C to completely remove residual methanol [61, 62].

#### 4.1.2 Printing of Liquid‐Metal Electrodes

A 1 µm‐thick bottom layer of parylene‐C was deposited via chemical vapor deposition to serve as the structural foundation. Liquid metal (i.e., EGaIn) electrodes were printed onto this layer using a 6‐axis pneumatic printing stage equipped with a 5 µm‐diameter nozzle. Printing conditions were precisely controlled (pressure, 50 psi; speed, 0.01 mm/s) to achieve electrode lines with a width of 5 µm. The electrode lengths were customized as 2, 4, 4.5, and 5 mm to accommodate varying locations of target brain regions.

A 2.5 µm‐thick top parylene‐C layer was then deposited to encapsulate the electrodes. Electrode tips were selectively opened using oxygen reactive ion etching (RIE) with a photoresist mask, creating 5 µm × 7.5 µm exposed areas. Final probe dimensions were defined using laser ablation (ProtoLaser U3, LPKF, Germany) to achieve a 20 µm‐width profile. Probes were carefully released from the wafer by applying a slight mechanical force at the tip.

#### 4.1.3 Electroplating of Platinum Nanoclusters

To enhance electrode performance, Pt nanocluster was electroplated onto the exposed tips. An electroplating solution was prepared by dissolving 0.5 g of Pt tetrachloride and 10 mg of lead acetate trihydrate in 50 mL of deionized water at room temperature. The mixture was homogenized using ultrasonic agitation for 20 min to ensure complete dissolution of reagents.

The soft neural probe served as the cathode, while a Ti/Pt sheet electrode was used as the anode. Both electrodes were immersed in the prepared solution and connected to a precision source meter (Keithley 2400). Electroplating was conducted by applying a constant current of 0.1 mA for 60 s, facilitating uniform deposition of Pt nanocluster onto the exposed electrode tips.

### 4.2 Calculating Bending Stiffness

The bending stiffness *D* of the multilayered neural probe (parylene‐C / EGaIn +Pt nanocluster / parylene‐C) was calculated using the following equation:

$$D=E_{eq}\;\cdot I_{x}$$

where *E_eq_* is the equivalent elastic modulus of the multilayer structure and *I_x_* is the moment of inertia. The equivalent modulus *E_eq_* was obtained as a thickness‐weighted average of the elastic modulus of each constituent layer:

$$E_{eq}=\sum_{i=1}^{n}\frac{E_{i}t_{i}}{t_{total}}$$

Here, *E_i_* and *t_i_* represent the elastic modulus and thickness of the *i*‐th layer, respectively, and *t_total_* is the total thickness of the multilayer structure. The Pt nanocluster was excluded from this calculation since it was selectively coated only at the tip of the electrode for recording purposes, and thus does not contribute to the overall bending stiffness.

The moment of inertia *I_x_* for a rectangular cross‐section with width *w* and thickness *t_total_* was calculated as:

$$I_{x}=\frac{w{\left(t_{total}\right)}^{3}}{12}$$

### 4.3 Characterization of Dissolution Profile of Methanol‐Treated Silk Fibroin

To characterize the dissolution profile of the silk fibroin stiffener, methanol‐treated silk fibroin samples deposited on glass substrates were immersed in 5 mL of a proteolytic solution at 37°C [61]. The proteolytic solution was prepared by dissolving Protease XIV (Protease XIV from Streptomyces griseus, Sigma‐Aldrich) in PBS at a concentration of 1 unit/mL, as silk fibroin is primarily degraded by proteolytic enzymes in the brain, and Protease XIV has been widely used to model extracellular proteolytic degradation under physiological conditions [84]. At 6 h intervals, samples were weighed after rinsing with ethanol and drying at 60°C for 10 min. To maintain consistent enzymatic activity, the proteolytic solution was refreshed after each measurement.

### 4.4 Cell Viability Test

SH‐SY5Y human neuroblastoma cells were cultured on the samples for 7 days, and cell viability was evaluated using a Live/Dead viability assay (Max‐view Live/Dead cell staining kit). For sample preparation, a 2 µm‐thick parylene‐C film was deposited on silicon wafers coated with a sacrificial layer (5 wt.% cellulose acetate in acetone), and silk fibroin films were drop‐cast on top. The silk fibroin layer was subjected to brief methanol treatment followed by vacuum drying at 40°C to remove residual methanol, consistent with the device fabrication process. Parylene‐C only films and methanol‐untreated silk fibroin samples were used as controls.

### 4.5 Animals

All surgical procedures were performed using adult C57BL/6 mice (Koatech). Animals were maintained in a specific pathogen‐free (SPF) facility with automated ventilation and water supply systems, under controlled environmental conditions (12‐hour light/dark cycle, 23°C, and 50% relative humidity). All experimental protocols were approved by the Yonsei University Institutional Animal Care and Use Committee and conducted in accordance with the Guide for the Care and Use of Laboratory Animals. All procedures were conducted in accordance with the guidelines in the Guide for the Care and Use of Laboratory Animals by Yonsei University Institutional Animal Care and Use Committee (IACUC‐A‐202404‐1844‐01).

### 4.6 Integration of Soft Neural Interface

One week after 6‐OHDA lesioning and Ad‐Piezo1 viral delivery, liquid metal‐based neural probes were implanted into mouse brain. Mice were anesthetized with an intraperitoneal injection of ketamine (100 mg/kg) and xylazine (25 mg/kg), and placed in a stereotaxic frame to ensure precise head positioning. The scalp was sterilized with iodine solution and incised to expose the cranium. After a thin (∼1 mm) layer of medical‐grade polymer (Loctite 4011, Henkel, Germany) covered the cranium, a small craniotomy was created at multiple target sites within the CBT circuit using a dental drill. The stereotaxic coordinates for each regions were as follows: Str (AP, +1.5 mm, ML, 1.6 mm, DV, ‐3.8 mm), GPe (AP, ‐0.34 mm, ML, 1.7 mm, DV, ‐3.5 mm), STN(AP, −2.06 mm, ML, 1.5 mm, DV, −4.5 mm), SNr (AP, −3.28 mm, ML, 1.5 mm, DV, −4.3 mm), Th (AP, −1.46 mm, ML, 0.8 mm, DV, −4.25 mm), and M1 (AP, +1.0 mm, ML, 1.5 mm, DV, −1.5 mm).

Sterilized probes (70% ethanol) were precisely inserted into each target brain region using a micromanipulator. Following insertion, liquid metal interconnects were directly printed onto the cranium surface using a 10 µm‐diameter nozzle. The printing was carried out at a constant pneumatic pressure of 36 psi, forming conductive lines approximately 8 µm in width. These interconnects were routed to a flexible flat connector to enable external interfacing. To ensure electrical insulation and prevent signal leakage, the printed interconnects were encapsulated with a soft silicon adhesive (Kwik‐sil, World Precision Instruments, USA). Following surgery, mice received appropriate analgesic treatment to alleviate pain and support recovery. A two‐week postoperative recovery period was allowed before initiating electrophysiological recordings or behavioral testing.

### 4.7 Impedance Spectroscopy

Impedance measurements were performed to evaluate the electrical properties of the EGaIn +Pt nanocluster electrodes. For in vitro impedance measurements, the electrodes were immersed in phosphate‐buffered saline (PBS; Sigma‐Aldrich) to simulate physiological conditions. For in vivo impedance measurements, the electrodes were implanted into mouse brain. Impedance spectra were obtained over a frequency range of 0.01 to 100 kHz using a multichannel potentiostat (PMC‐1000, AMETEK).

### 4.8 Accelerated Aging Test

The duration of the accelerated aging test was determined using the following equation, which relates the accelerated aging duration to the desired real‐time equivalent based on a temperature‐acceleration model:

$$Accelerated\;aging\;duration=Desired\;real\;time\;\times Q_{10}^{-\left(T_{AA}-T_{AMB}\right)/10}$$

where Q_10_ represents the aging acceleration factor (∼2), T_AA_ is the accelerated aging temperature (74°C), and T_AMB_ is the ambient temperature [85]. For a target storage period of 6 months, T_AMB_ was assumed to be the physiological temperature (37°C), resulting in a calculated accelerated aging duration of approximately 8 days.

### 4.9 Immunohistochemistry

For immunohistochemical analysis, mice were deeply anesthetized by intraperitoneal injection of ketamine (100 mg/kg) and xylazine (25 mg/kg), followed by transcranial perfusion with phosphate‐buffered saline (PBS; Sigma–Aldrich) and subsequently with 4% paraformaldehyde (PFA; Sigma‐Aldrich) in PBS. Brains were extracted and post‐fixed in the same fixative for 24 h at 4°C, cryoprotected in 30% sucrose solution, and serially sectioned at a thickness of 40 µm using a microtome (Leica Microsystems GmbH). Brain sections were stored in cryoprotectant solution at 4°C until further use.

Free‐floating brain sections were blocked for 1 h at room temperature in PBS containing 3% bovine serum albumin (BSA), 5% normal goat serum, and 0.1% Triton X‐100 to prevent nonspecific binding. Sections were then incubated overnight at 4°C with primary antibodies diluted in blocking solution: anti‐Myc (Cell Signaling Technology, 1:500), anti‐c‐Fos (Abcam, 1:500), anti‐Iba1 (Abcam, 1:500), anti‐GFAP (BioLegend, 1:500), and anti‐tyrosine hydroxylase (Abcam, 1:1000).

After several washes with PBS, sections were incubated with appropriate secondary antibodies for 3 h at room temperature: anti‐rabbit IgG (Alexa Fluor 647, Abcam, goat polyclonal, 1:500), anti‐mouse IgG (Alexa Fluor 488, Abcam, goat polyclonal, 1:500), or anti‐chicken IgG (Alexa Fluor 488, Abcam), followed by nuclear counterstaining with DAPI (Invitrogen). Sections were then washed, mounted, and imaged using a confocal microscope (Zeiss, Germany). Fluorescence images were acquired using ZEN software and analyzed with ImageJ.

### 4.10 X‐Ray Imaging

X‐ray imaging was performed to verify the accurate implantation and positioning of neural probes within the brain. C57BL/6N mice were anesthetized using an appropriate anesthetic regimen and placed in a stereotaxic apparatus to ensure stable head positioning. After probe implantation, the mice were perfused with 20 mL of PBS (Sigma‐Aldrich) to clear the blood, followed by perfusion with paraformaldehyde (PFA; Sigma‐Aldrich) to fix the brain tissue. The brains were then imaged using an X‐ray imaging system (AXIS‐1130, Amfis X‐ray).

### 4.11 Synthesis and Functionalization of 200 nm m‐Torquer

The 200 nm m‐Torquer was synthesized by chemically assembling magnetic nanoparticles (MNPs) onto 200 nm polystyrene (PS) supports, following a previously reported method with minor modifications [27]. Octahedral zinc‐doped iron oxide MNPs were synthesized via a thermal decomposition method [86]. Briefly, 1.65 mmol of tris(2,4‐pentanedionato)iron(III) (TCI), 1.65 mmol of zinc 2,4‐pentanedionate monohydrate (Alfa Aesar), 2.2 mL of oleic acid (Sigma‐Aldrich), and 10 mL of benzyl ether (Sigma‐Aldrich) were mixed in a three‐neck round‐bottom flask. The temperature was ramped from room temperature to 290°C over 50 min, followed by a 1‐hour hold at 290°C. The reaction mixture was cooled to room temperature, and the resulting product was washed several times via centrifugation using isopropanol and toluene. The purified MNPs were suspended and stored in toluene.

To introduce functional groups, MNPs were first encapsulated in amine‐functionalized silica. Azide groups were subsequently introduced by reacting 50 µmol of azido‐dPEG_12_‐TFP ester (Quantabio) with 1 nmol of amine‐functionalized MNPs in DMSO, with shaking for 4 h at room temperature. The azide‐functionalized MNPs were purified by centrifugation and redispersed in DMSO. Separately, DBCO‐functionalized 200 nm polystyrene beads were prepared by reacting 0.7 µmol of DBCO‐PEG_5_‐TFP ester (Click Chemistry Tools) with 0.2 pmol of amine‐functionalized PS microspheres (Polysciences) in DMSO, also under shaking for 4 h at room temperature. The beads were then purified and resuspended in DMSO. The azide‐functionalized MNPs were conjugated to the DBCO‐functionalized PS supports at a molar ratio of 2000:1 (MNP:PS) in DMSO. After 8 h of incubation at room temperature, unbound MNPs were removed by centrifugation. For further surface functionalization, carboxylate groups were introduced using DBCO‐PEG_5_‐COOH (Click Chemistry Tools) and succinic anhydride (Sigma‐Aldrich), each at a final concentration of 1 mg/mL. The resulting carboxylated m‐Torquer was purified via centrifugation and resuspended in 1 mM phosphate buffer (pH 7.2).

To conjugate Myc antibody on the m‐Torquer surface, 385.67 µg of Protein A (Sigma–Aldrich) was first coupled to 1 mg of carboxylated m‐Torquer via EDC/NHS chemistry, followed by purification via centrifugation. Then, 113.87 µg of Myc antibody (Sigma‐Aldrich) was conjugated to the Protein A‐bound m‐Torquer. The final product was purified and resuspended in 1 mM phosphate buffer (pH 7.2) at a final concentration of 50 mg/mL.

### 4.12 Plasmid and Viral Vector Construction

The pcDNA3.1‐Piezo1‐IRES‐eGFP plasmid was generously provided by Dr. Ardem Patapoutian (The Scripps Research Institute, La Jolla, CA, USA). For in vivo magnetogenetic stimulation experiments, the coding sequence of mouse Piezo1 with a Myc epitope tag inserted at position 897 (Myc897‐Piezo1) was subcloned into the pENTCMV vector, which is based on a human adenovirus serotype 5 backbone lacking E1 and E3 regions (ΔE1/E3; KOMABIOTECH, South Korea). The resulting recombinant adenovirus (Ad‐Piezo1) was produced at a final concentration of 1 × 10^1^ ^2^ virus particles per milliliter (VP/ml) using standard viral packaging protocols.

### 4.13 Animal Surgery for 6‐OHDA Injection and Ad‐Piezo1 Delivery

Mice were anesthetized with a mixture of ketamine (100 mg/kg) and xylazine (25 mg/kg), and placed in a stereotaxic frame (David Kopf Instruments). For the induction of PD, 6‐OHDA (4 µg/µL in 0.9% saline with 0.02% ascorbic acid) was injected unilaterally into the medial forebrain bundle (MFB; coordinates from bregma: AP, −1.20 mm; ML, 1.20 mm; DV, −4.75 mm) using a microsyringe pump system (Micro4, WPI) with a 10 µL Nanofil syringe fitted with a 33‐gauge needle. The injection volume was 0.9 µL at a rate of 0.3 µL/min. The needle was left in place for 5 min post‐injection to ensure proper diffusion and prevent backflow. For sham‐operated controls, the same surgical procedures were followed, except that saline was injected into the MFB instead of 6‐OHDA.

On the same day, a cocktail composed of recombinant adenoviral vectors encoding Myc‐tagged Piezo1 (Ad‐Piezo1; 0.75 µL, 1 × 10^1^ ^2^ VP/mL) and magnetic nanoparticle (m‐Torquer; 0.75 µL, 50 mg/mL) was unilaterally injected into the STN (AP, ‐2.06 mm; ML, 1.50 mm; DV, −4.50 mm) at a total volume of 1.5 µL. The injection was performed at a rate of 0.3 µL/min using the same stereotactic setup. Following injection, the needle was maintained in place for 5 min to ensure efficient delivery.

Postoperative care included daily monitoring, and mice exhibiting signs of dehydration were subcutaneously administered 200 µL of sterile glucose‐saline solution for up to 7 days. All animals recovered on a heating pad and were monitored for any signs of distress or complications.

### 4.14 Electrophysiological Recordings

Electrophysiological signals were recorded using a system comprising an RZ2 amplifier processor, PZ5 Neurodigitizer, and MZ60 MEA interface (Tucker‐Davis Technologies Inc., USA), controlled by a computer running the Synapse software. Signals were sampled at a rate of 24,414 Hz with a 60 Hz notch filter applied during recording to eliminate line noise. For local field potential (LFP) recordings, a 0.1–300 Hz bandpass filter was primarily used to isolate relevant frequency bands.

Recordings were conducted for 2 min each immediately before and after MG‐DBS application. During recording, the mice were anesthetized and securely head‐fixed in a stereotaxic apparatus to minimize motion artifacts. The entire setup was placed inside a Faraday cage to eliminate external electrical interference, ensuring high‐quality signal acquisition.

### 4.15 Setup and Operation of MG‐DBS

The MG‐DBS system was developed using a custom‐designed rotational magnetic force generator (MFG). The MFG consisted of a circular array of 1 T neodymium‐iron‐boron (NdFeB) magnets, configured to produce a uniform magnetic field with minimal spatial gradients. The entire MFG unit, with a diameter of 16 cm, was mounted on a motorized rotation stage constructed in‐house and controlled via an Arduino‐based system. The rotation system was programmed to support bidirectional motion (clockwise and counterclockwise) with adjustable speed, enabling fine control over stimulation parameters. To ensure spatial uniformity and field strength, the magnetic field distribution was simulated using finite element analysis (FEA) in COMSOL Multiphysics.

For in vivo stimulation, mice were placed in a 16 cm‐diameter circular arena positioned directly atop the rotating MFG platform. During MG‐DBS, the system generated a uniform magnetic field with an amplitude of approximately |B|  =  52.6 mT at a rotational frequency of 0.5 Hz. These stimulation parameters enabled consistent and reproducible magneto‐mechanical neuromodulation. Mice were subjected to MG‐DBS stimulation for 30 min per session under these conditions.

### 4.16 Open Field Test

The open field test was conducted to evaluate the locomotor activity in Parkinsonian mice. The test was performed in a 1200 cm^2^ square arena. The mice were placed in the center of the arena and allowed to move freely for 5 min. Mouse movements were video‐recorded and analyzed using ANY‐maze (version 4.3; Stoelting Co., Wood Dale, IL) and Tracker (version 6.3.0) software. Locomotor activity was measured as the total distance traveled within the arena.

### 4.17 Electrical Stimulation

For electrical stimulation, Pt‐Ir electrodes (inner diameter: 50.8 µm; A‐M Systems) were stereotaxically implanted into the STN (AP, −2.06 mm; ML, 1.50 mm; DV, −4.50 mm) concurrently with 6‐OHDA lesioning. The electrodes were secured to the skull using dental cement to ensure stability during stimulation. After a three‐week recovery period, electrical stimulation was delivered to the STN at 125 Hz with a current amplitude of 200 µA and a pulse width of 60 µs.

### 4.18 Statistical Analysis

All data are presented as mean ± standard error of the mean (SEM). Statistical significance is indicated by *(*p* <0.05), **(0.01), **(0.001), ***(0.0001), and ****(0.00001) in the figures and figure legends. For Figure 2, the normalized power was calculated by dividing the power of each frequency band by the total power integrated over 0.1–100 Hz. Sample size are provided in the corresponding figure captions. Electrophysiological data were analyzed using MATLAB software.

## Funding

This work was supported by the Ministry of Science & ICT (MSIT), the Ministry of Trade, Industry and Energy (MOTIE), the Ministry of Health & Welfare, and the Ministry of Food and Drug Safety of Korea through the National Research Foundation (RS‐2023‐NR077138, RS‐2024‐00464032, RS‐2025‐16063568, RS‐2025‐18362970), STEAM Research Programs (RS‐2024‐00460364), ERC Program (RS‐2024‐00406240), and Korea Institute of Science and Technology (KIST) Institutional Program (2E33191 and 2E33190). The authors thank the financial support by the Institute for Basic Science (IBS‐R026‐D1).

## Conflicts of Interest

The authors declare no conflicts of interest.

## Supporting information




**Supporting File 1**: adhm71129‐sup‐0002‐SuppMat.pdf.

**Supporting File 2**: adhm71129‐sup‐0001‐MovieS1.mp4.

## Data Availability

The data that support the findings of this study are available from the corresponding author upon reasonable request.
