# Supplementary material for: Soft Neural Interfaces for Circuit‐Level Analysis of Magnetogenetic Deep Brain Stimulation in Parkinson's Disease Models
Source: Adv Healthc Mater. 2026 Apr 15;15(22):e05548. doi: 10.1002/adhm.202505548 (PMC13279859; doi:10.1002/adhm.202505548)
Supplement: Supplementary file 1 — Supporting File 1: adhm71129‐sup‐0002‐SuppMat.pdf. [file ADHM-15-0-s001.pdf]

Supporting Information

Soft neural interfaces for circuit-level magnetogenetic deep brain stimulation in Parkinson's disease models

Jakyoung Lee, Yeongdo Lee, Enji Kim, Hunkyu Seo, Myoungjae Oh, Hanho Joo, Somin Lee, Jae-Hyun Lee, Hyun Ho Jung, Minsuk Kwak, Jang-Ung Park**

This supporting information file includes:

Note S1

Figure S1-30

Captions for Movie S1

Supporting References

Supporting Note

Note S1. Mechanical modeling of buckling force and required silk fibroin thickness to achieve reliable insertion

To ensure reliable insertion of the liquid metal-based soft neural probe into mouse brain tissue, we conducted a mechanical analysis to estimate the minimum thickness of the silk fibroin stiffener required to prevent buckling. Prior studies have indicated that a buckling force of 1 mN is necessary for successful penetration into rodent brain tissue.[1–3]

We modeled the buckling force for our four-layered probe structure composed of silk fibroin / parylene-C / EGaIn + Pt nanoclusters / parylene-C. The buckling force ($F_{buckling}$) of the entire structure is defined as equation (1),

$$F_{buckling} = \frac{\pi^2 I_x E_{eq}}{(KL)^2} \quad (1)$$

where I_x is the moment of inertia, E_{eq} is the equivalent elastic modulus of the entire structure, K is the effective length factor, and L is the length of the probe.

Given that the number of structure layers was fewer than 100, the effect of shear interaction between layers was considered negligible.[4] Accordingly, we assumed that the probe exhibits mechanical behavior similar to that of a single homogeneous rectangular beam. Based on this assumption, the moment of inertia and equivalent elastic modulus were calculated using the following equations (2) and (3), respectively,

$$I_x = \frac{w(t_{total})^3}{12} \quad (2)$$

$$E_{eq} = \sum_{i=1}^{n} \frac{E_i t_i}{t_{total}} \quad (3)$$

where w is the probe width (20 μm), E_i and t_i are the elastic modulus ($E_{silk\ fibroin}$ = 3 GPa, $E_{parylene\text{-}C}$ = 2.9 GPa, E_{EGaIn} = 200 kPa) and the thickness ($t_{parylene\text{-}C}$ = 3.5 μm, t_{EGaIn} = 2.5 μm) and of each individual layer, and t_{total} is the total thickness of the probe.[5-8] As Pt nanoclusters were only deposited onto the probe tip, their mechanical contribution was excluded from the calculation.

The structure was considered as a beam model with one fixed end, and an effective length factor of K = 0.707 was applied accordingly.[9] To ensure a conservative estimate, we selected the longest probe length among our designs (L = 5 mm).

Using these parameters, we calculated the buckling force for probes with silk fibroin thickness of 40, 45, 50, 55, and 60 μm. The results are presented in Figure S5. Based on this analysis, we determined that a silk fibroin thickness of approximately 58.2 μm is required to exceed the 1 mN buckling threshold.

Supporing Figures

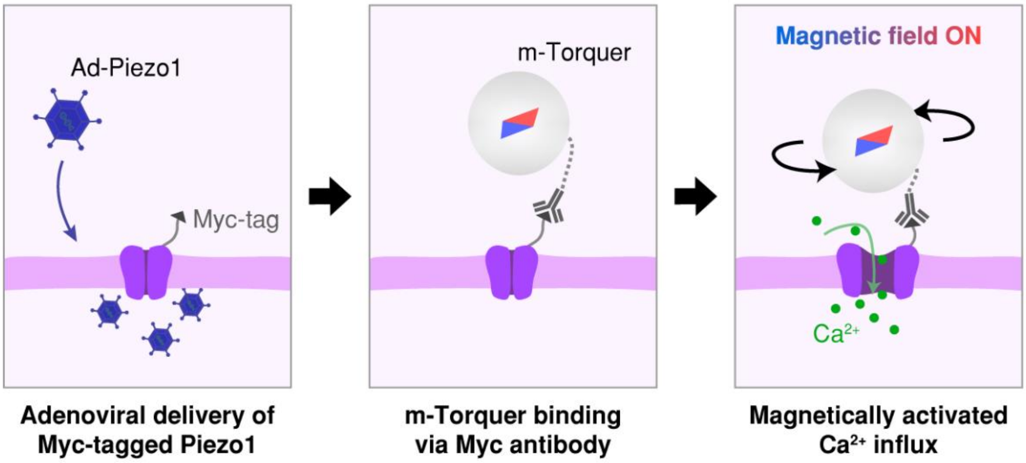

Figure S1. Schematic illustration of magnetogenetic deep brain stimulation (MG-DBS) process using Ad-Piezo1 and m-Torquer.

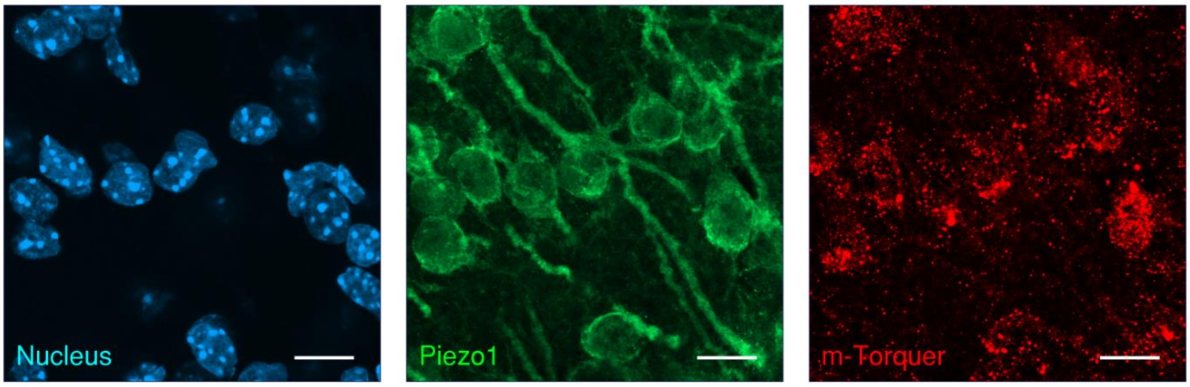

Figure S2. Immunofluorescence images showing cell nuclei stained with DAPI (blue, left), Piezo1 expression (green, middle), and distribution of m-Torquer (red, right) in the same field of view. Scale bars, 10 μm.

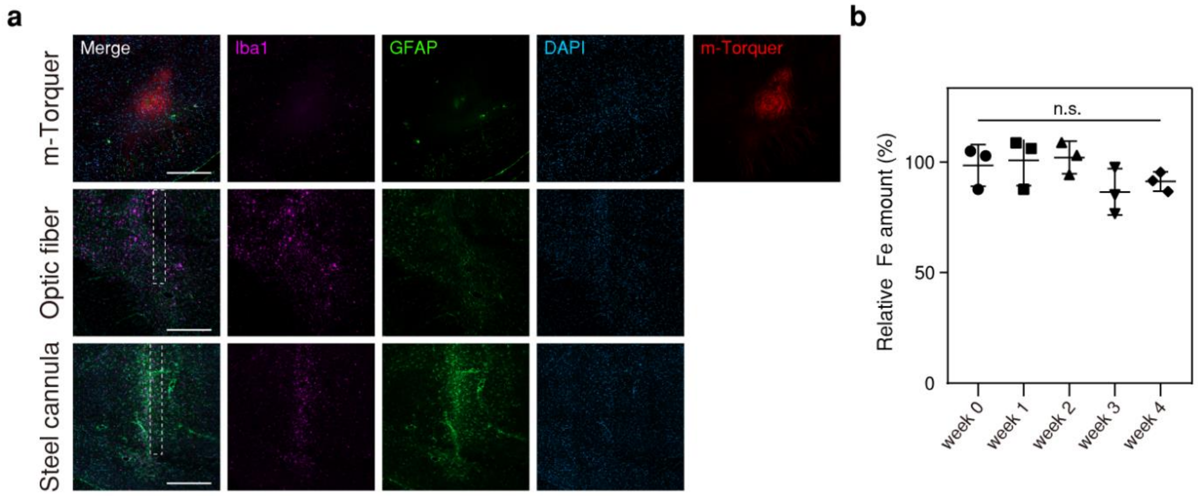

Figure S3. Biocompatibility of m-Torquer implantation in the subthalamic nucleus (STN). **a**, Representative immunofluorescence images of STN sections obtained 3 weeks after implantation of m-Torquer, optic fiber, or steel cannula. Sections were stained for GFAP (astrocytes, green), iba1 (microglia, magenta), and nuclei (DAPI, blue), with m-Torquer distribution visualized in red. Compared to conventional implants (optic fiber and steel cannula), minimal glial activation is observed around the m-Torquer, indicating reduced neuroinflammatory response. Scale bars, 300 µm. **b**, Quantification of relative iron (Fe) content in the brain at 0-, 1-, 2-, 3- and 4-week post-implantation. No significant difference (n.s., p = 0.2932) was detected over time, suggesting stable long-term retention of m-Torquer without substantial degradation.

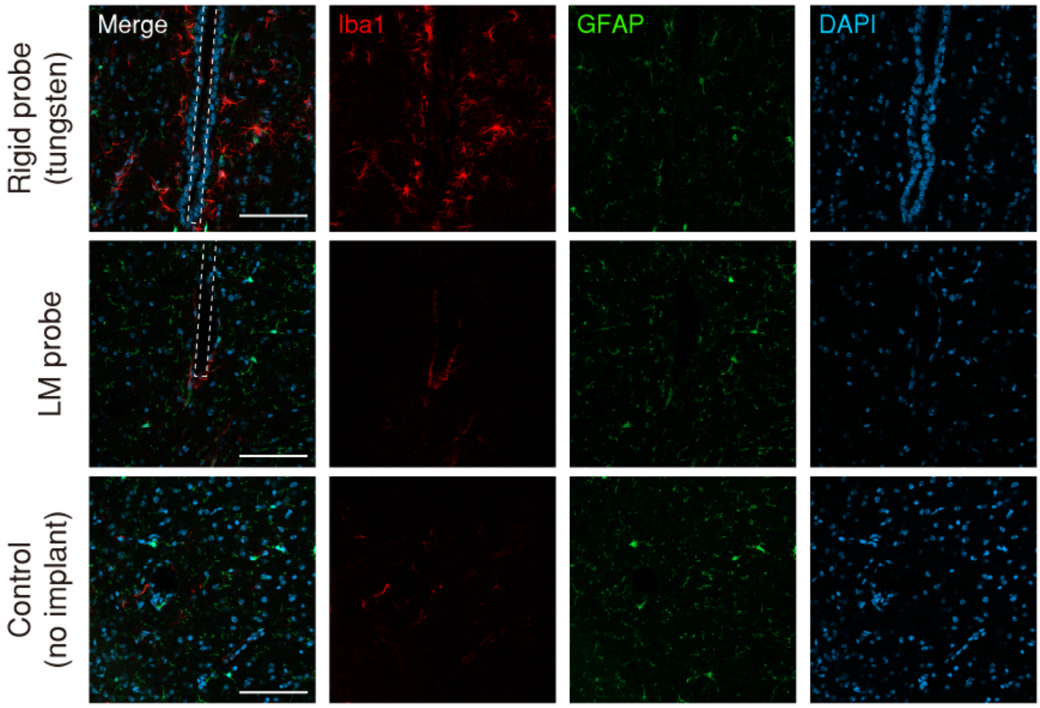

Figure S4. Immunofluorescence images of STN brain sections obtained 6 weeks after implantation of rigid tungsten probe or a soft LM probe, with a no-implant group as control. Sections were stained for iba1 (microglia, magenta), GFAP (astrocytes, green), and nuclei (DAPI, blue). Dashed outlines indicate the probe tracks. Scale bars, 100 µm.

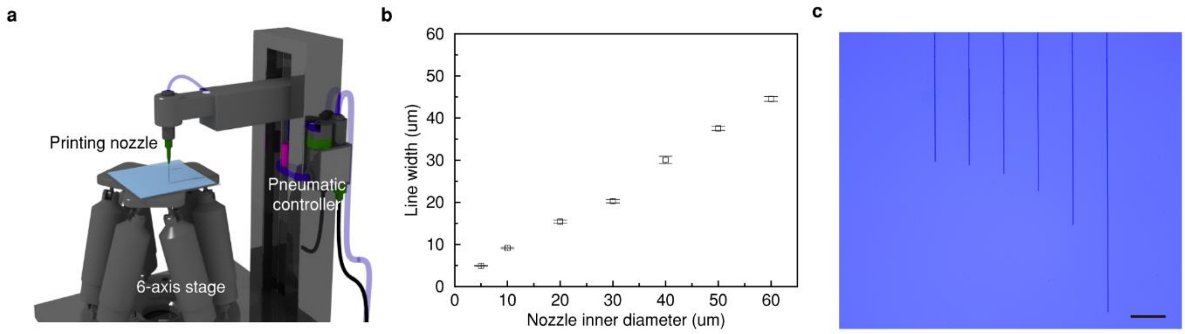

Figure S5. High-resolution liquid-metal (LM) printing system. **a**, Schematic illustration of a 6-axis printing system comprising a pneumatic controller, printing nozzle, and 6-axis precision stage. **b**, Relationship between nozzle inner diameter and resulting line width of printed LM. Data are presented as mean ± s.e.m.. **c**, Optical image of LM lines printed at various lengths. Scale bar, 200 μm.

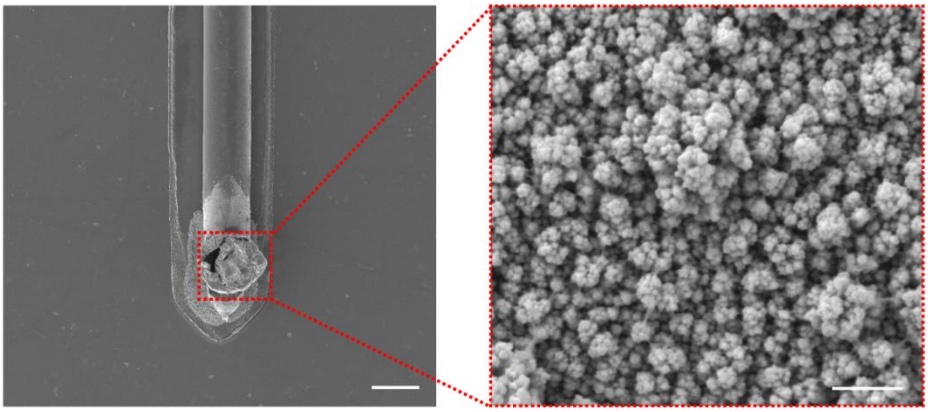

Figure S6. Scanning electron microscopy (SEM) images of the LM-based neural probe (left), and magnified view of platinum (Pt) nanoclusters electroplated on the probe tip (right). Scale bars, 5 µm (left), 500 nm (right).

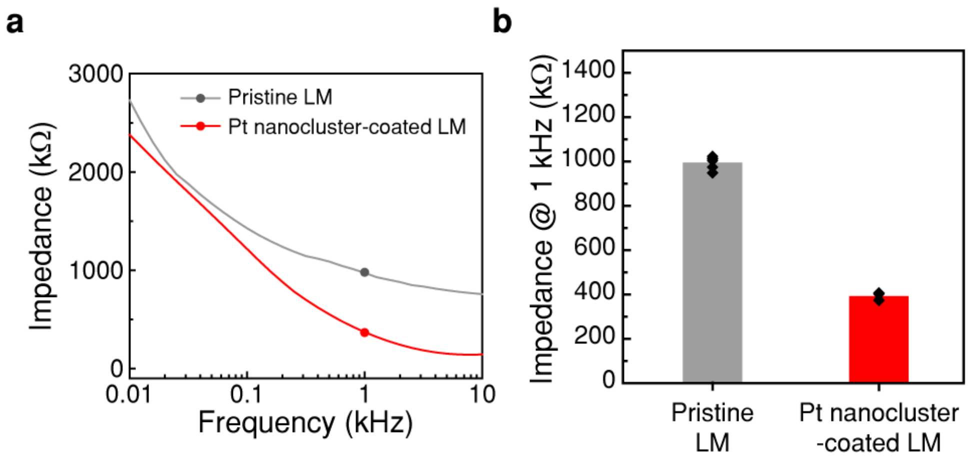

Figure S7. Impedance characterization of LM-based electrodes. **a**, Representative impedance spectroscopy of the LM electrode coated with Pt nanoclusters (red) and pristine LM electrode (grey). The impedance value at 1 kHz is marked with dots. **b**, Bar graph summarizing the impedance values at 1 kHz for each electrode. Data are presented as mean ± s.e.m..

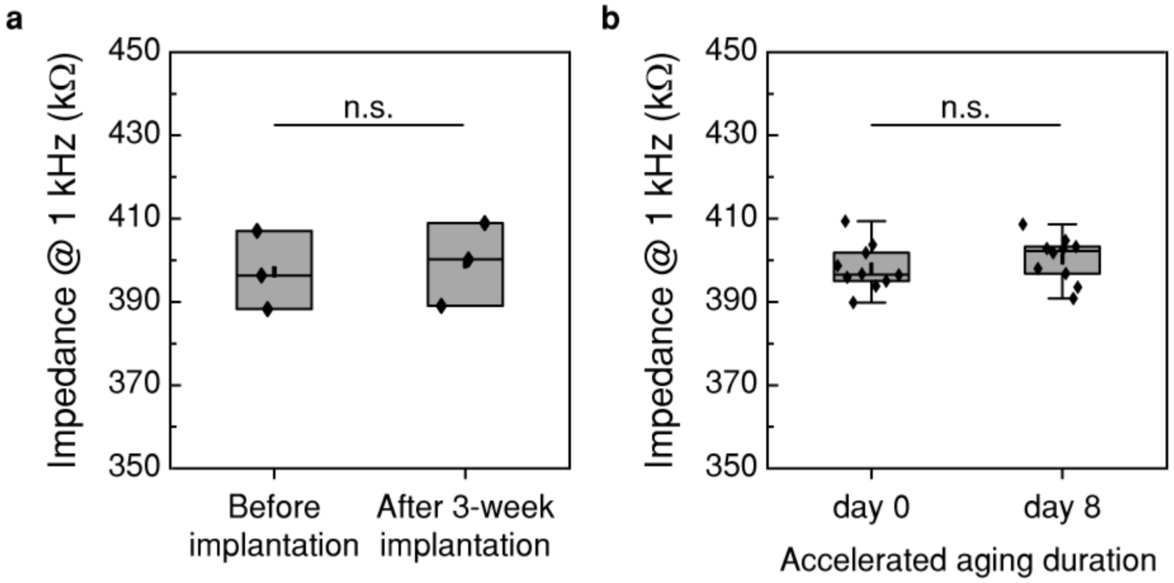

Figure S8. Chronic impedance stability of Pt nanocluster-coated LM electrodes. **a**, Impedance measured at 1 kHz before implantation and 3 weeks after implantation followed by explantation. **b**, Impedance measured at 1 kHz under accelerated aging test in PBS at 74°C (corresponding to 6 months under physiological conditions). No significant changes were observed (n.s.).

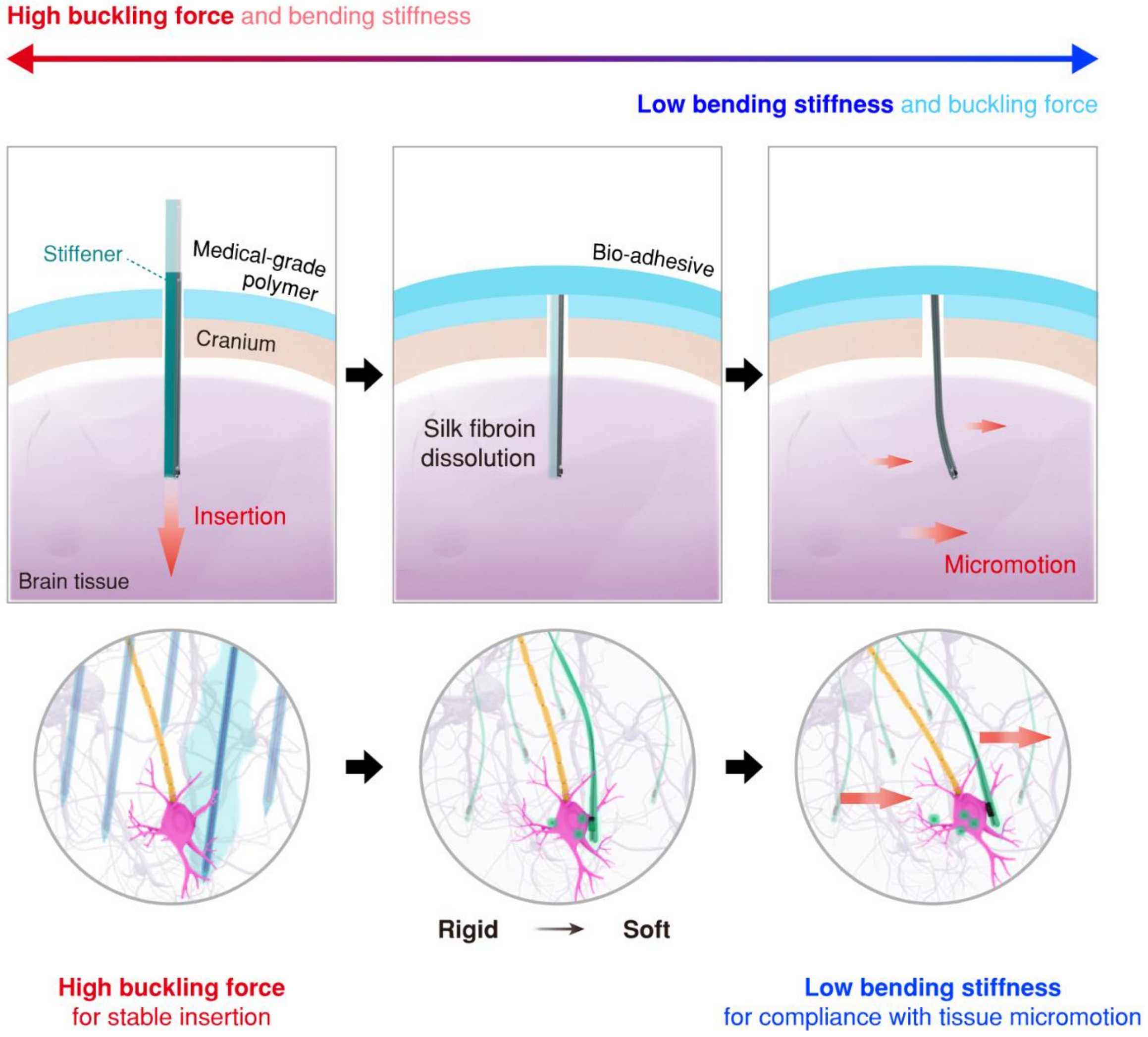

Figure S9. Schematic illustrations describing mechanical behavior of the soft neural probe integrated with a stiffener during implantation and within the brain tissue.

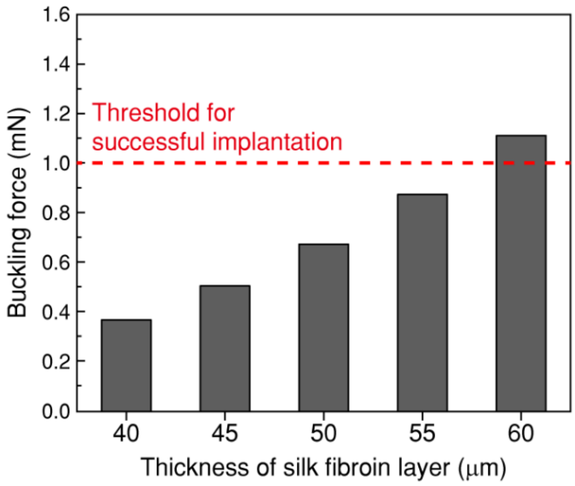

Figure S10. Calculated buckling force as a function of silk fibroin thickness. Red dotted line represents the threshold buckling force (1 mN) required for successful implantation into mouse brain.

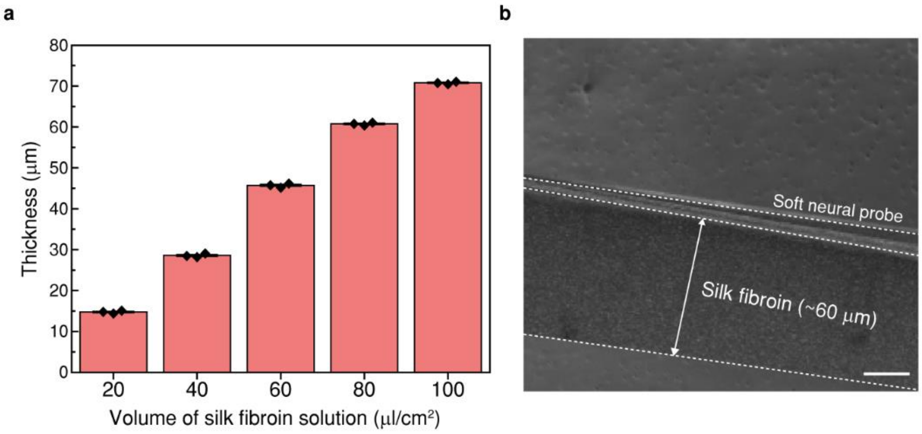

Figure S11. Optimization of silk fibroin casting volume to achieve target stiffener thickness. **a**, Relationship between the volume of 10 wt.% silk fibroin solution (per 1 cm^2 area) and the resulting film thickness. **b**, Cross-sectional SEM image of the soft neural probe integrated with a silk fibroin stiffener (~60 μm thick). Scale bar, 20 μm.

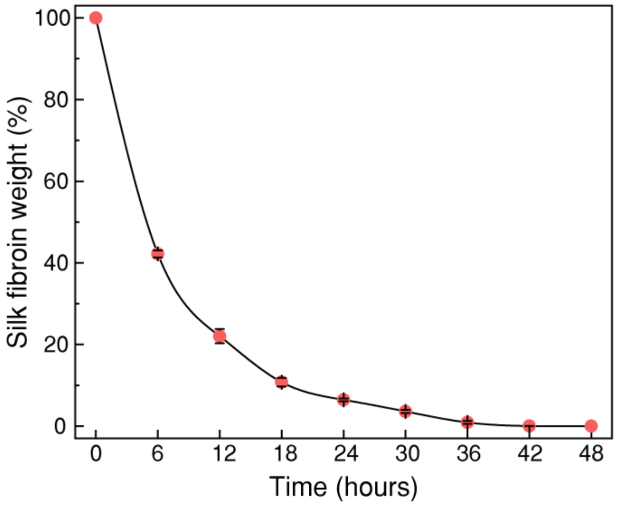

Figure S12. Dissolution profile of methanol treated silk fibroin layer. Each sample was immersed in a proteolytic solution (1 unit/mL protease XIV in PBS) at 47°C and weighed every 6 h after rinsing with ethanol and drying at 60°C for 10 min. Data are presented as mean ± s.e.m..

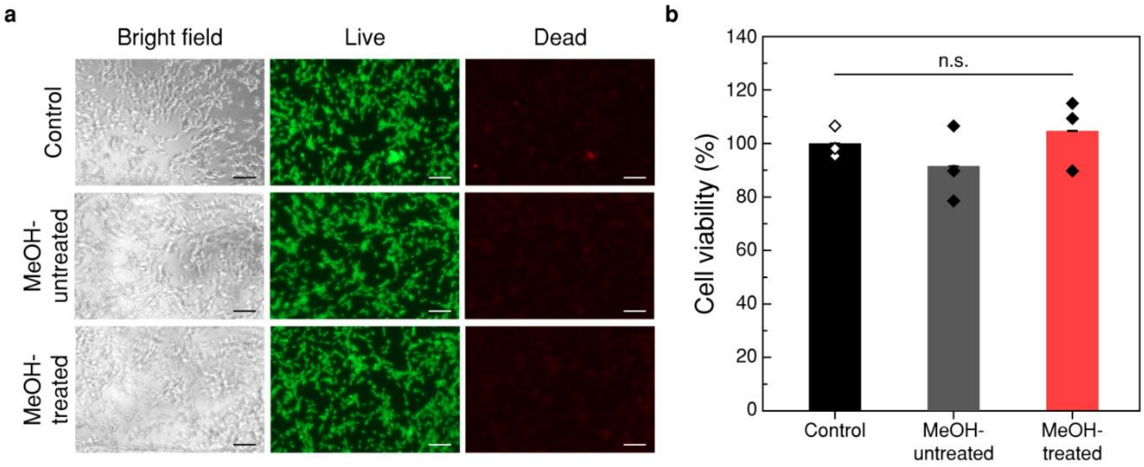

Figure S13. Cell viability test of methanol-treated sample. **a**, Live/Dead staining of SH-SY5Y human neuroblastoma cells cultured for 7 days on a 2 μm-thick parylene-C film without silk fibroin (Control), with pristine silk fibroin (MeOH-untreated), and with methanol-treated silk fibroin (MeOH-treated). Scale bars, 100 μm. **b**, Quantification of Live/Dead assay after 7 days of culture.

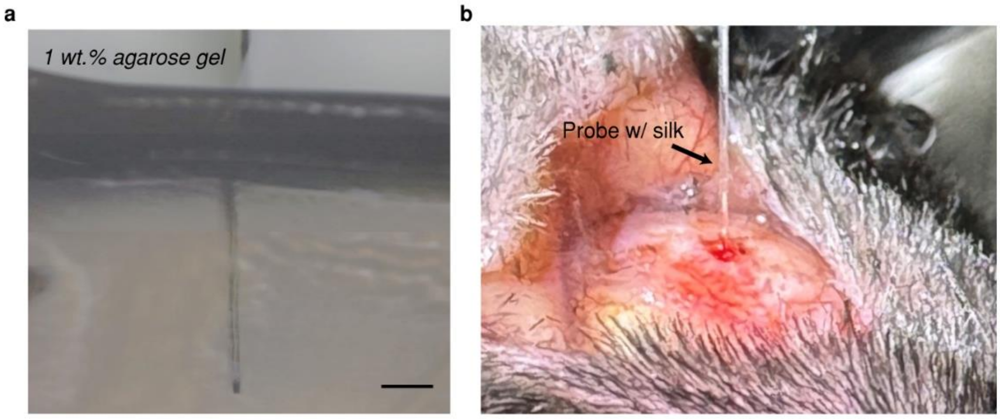

Figure S14. a, Insertion performance of the silk fibroin integrated neural probe into1 wt.% agarose gel. Scale bar, 500 µm. **b**, Successful insertion of the probe into the mouse brain.

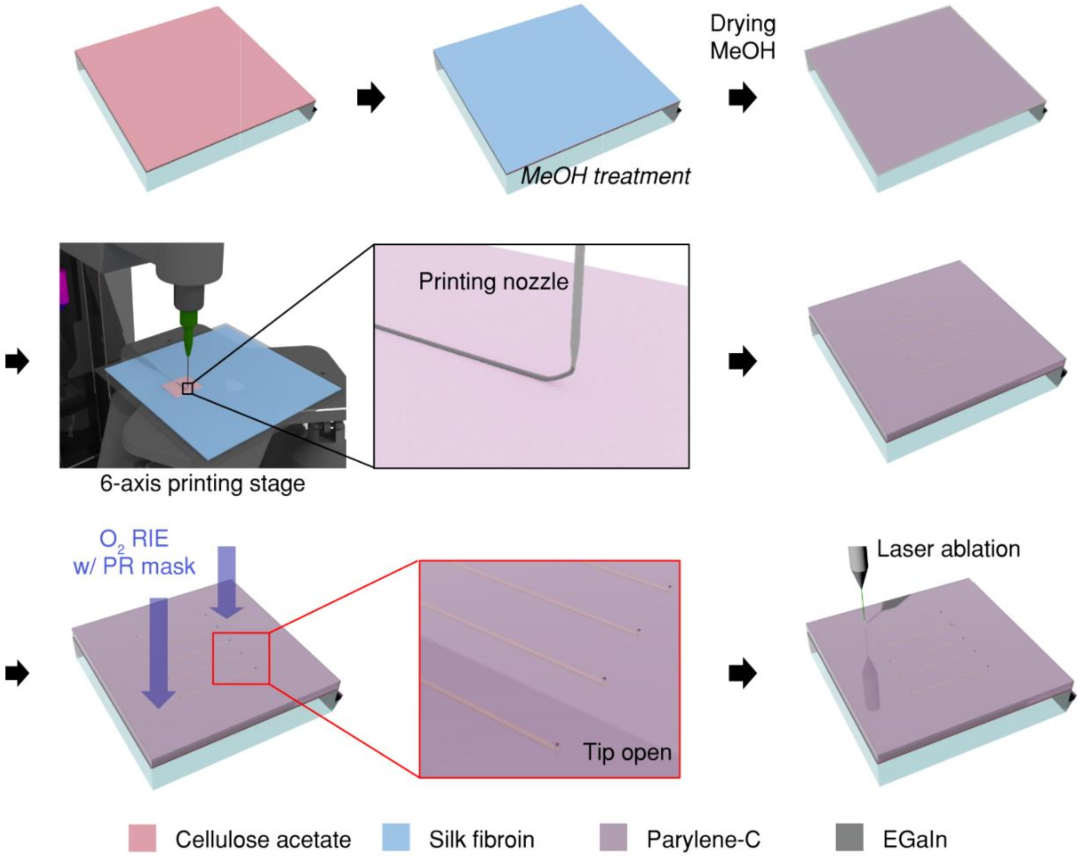

Figure S15. Schematic illustrations of the fabrication process for the LM-based soft neural probe.

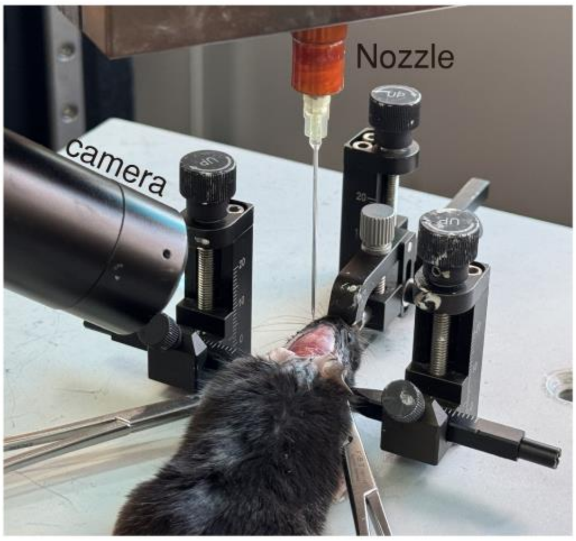

Figure S16. Photograph of direct printing of LM interconnections onto the mouse cranium using the high-resolution printing system.

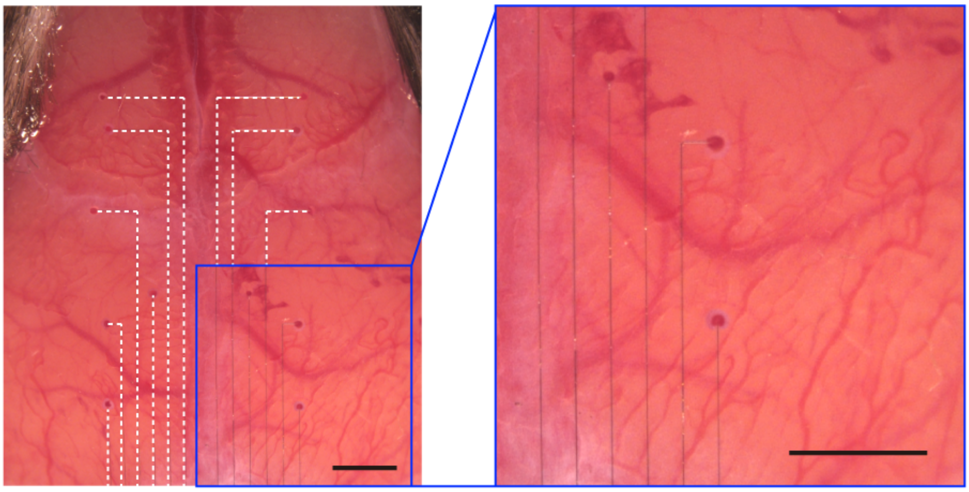

Figure S17. Optical stereomicrographs of LM-based interconnections printed onto cranial surface, as indicated by the white dashed outlines (left) and a magnified view of the blue boxed region (right). Scale bars, 1 mm.

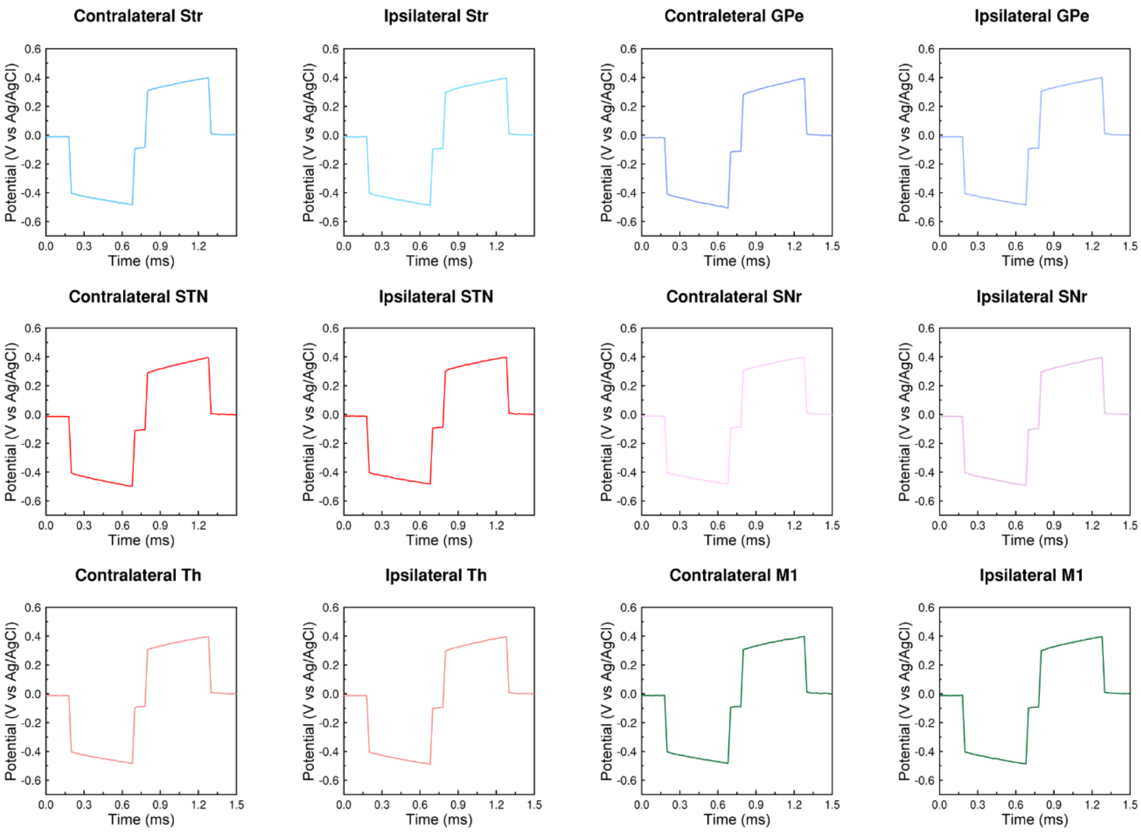

Figure S18. Electrical waveforms recorded from twelve individual LM-based interconnection electrodes in response to biphasic current (0.5 mA amplitude, 0.5 ms pulse width).

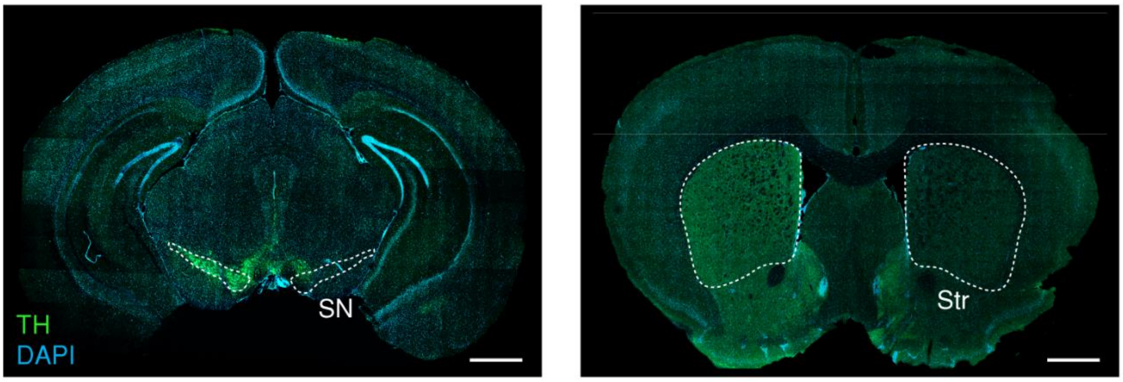

Figure S19. Immunofluorescence images of coronal brain sections from a mouse model of unilateral 6-hydroxydopamine (6-OHDA) lesion. Dopaminergic neurons were labeled with tyrosine hydroxylase (TH, green), and cell nuclei were counterstained with DAPI (blue). Both the substantia nigra (SN, left) and striatum (Str, right) regions are visible, with reduced TH expression observed in the ipsilateral hemisphere. The Str and SN regions are outlined with dashed lines. Scale bar, 1 mm.

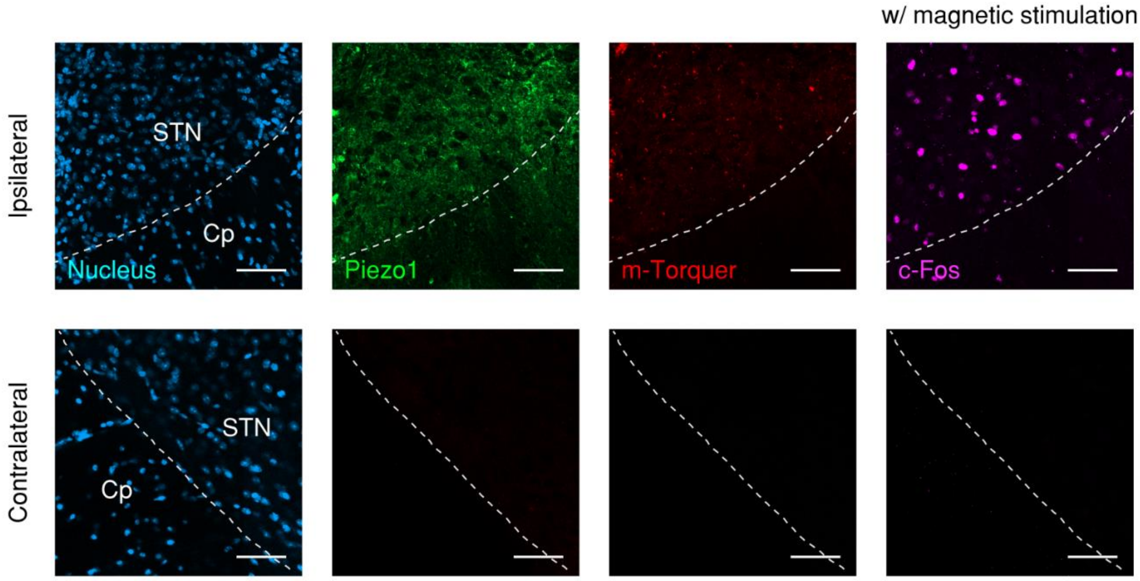

Figure S20. Immunofluorescence images of the ipsilateral (top) and contralateral (bottom) subthalamic nucleus (STN), showing cell nuclei (DAPI, blue), Piezo1 expression (green), m-Torquer distribution (red), and c-Fos induction following magnetic stimulation (pink). c-Fos expression is observed only in ipsilateral hemisphere, indicating localized neuronal activation. Scale bars, 50 µm. Cp, cerebral peduncle.

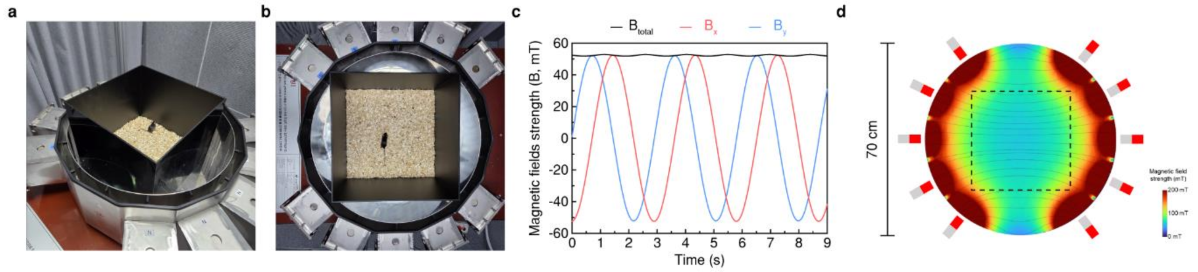

Figure S21. Experimental setup for MG-DBS in vivo. **a**,**b**, Photographs of the rotational magnetic stimulation system with a 70 cm-diameter arena surrounded by 10 permanent magnets, shown from a side view (**a**) and a top view (**b**). **c**, Temporal profiles of the magnetic field components in the x (red) and y (blue) axes, and the resultant total magnetic field magnitude (black), showing a constant amplitude of 52.6 mT. **d**, COMSOL simulation of the magnetic field magnitude (|B|) in the 70 cm-diameter arena. The 40 x 40 cm square arena is indicated by the black dotted square.

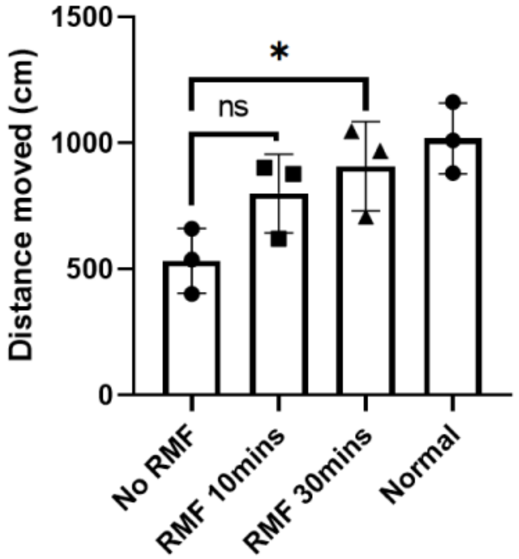

Figure S22. Behavioral effects of rotating magnetic field stimulation depend on stimulation duration. Locomotor activity was assessed by measuring total distance moved in Parkinsonian mice exposed to rotating magnetic field stimulation for different durations. 10 min stimulation did not induce a statistically significant behavioral improvement compared with the no rotating magnetic field condition (n.s.), 30 min stimulation resulted in a significant increase in locomotor activity (*$p < 0.05$).

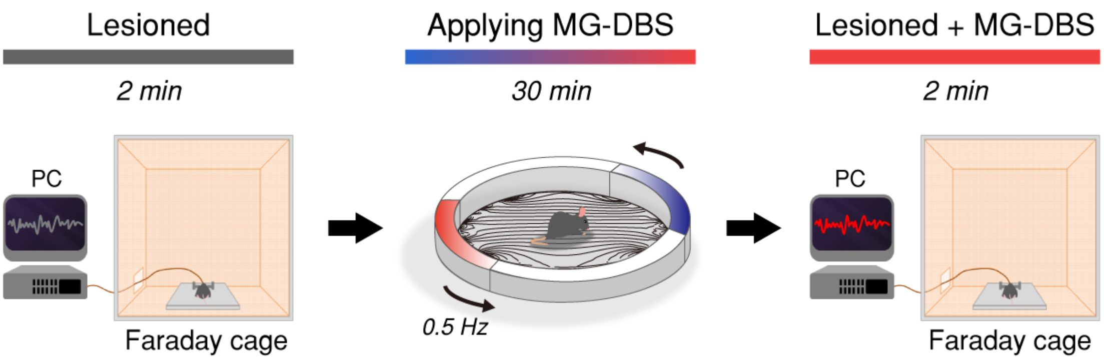

Figure S23. Schematic illustration of the experimental procedure for electrophysiological recordings and MG-DBS.

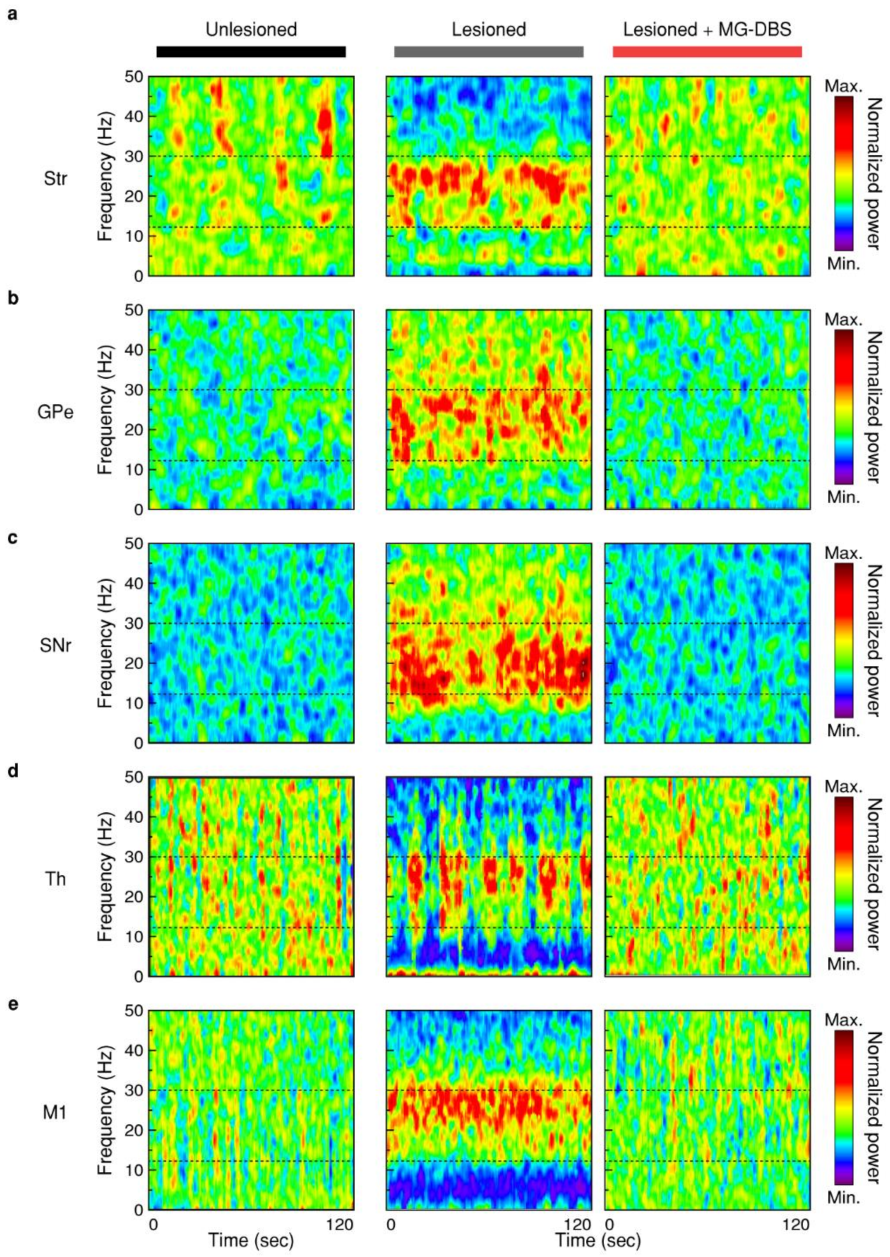

Figure S24. Time-frequency spectrogram of LFPs across multiple brain regions. **a-e**, Representative power spectrograms of LFP recorded from Str (**a**), GPe (**b**), SNr (**c**), Th (**d**), and M1 (**e**) under three conditions: unlesioned hemisphere (left), lesioned hemisphere (middle), and lesioned hemisphere with MG-DBS (right). Each spectrogram displays the normalized spectral power across the 0.50 Hz frequency range during a 2-minute recording period. Dashed lines mark the beta band range (12-30 Hz).

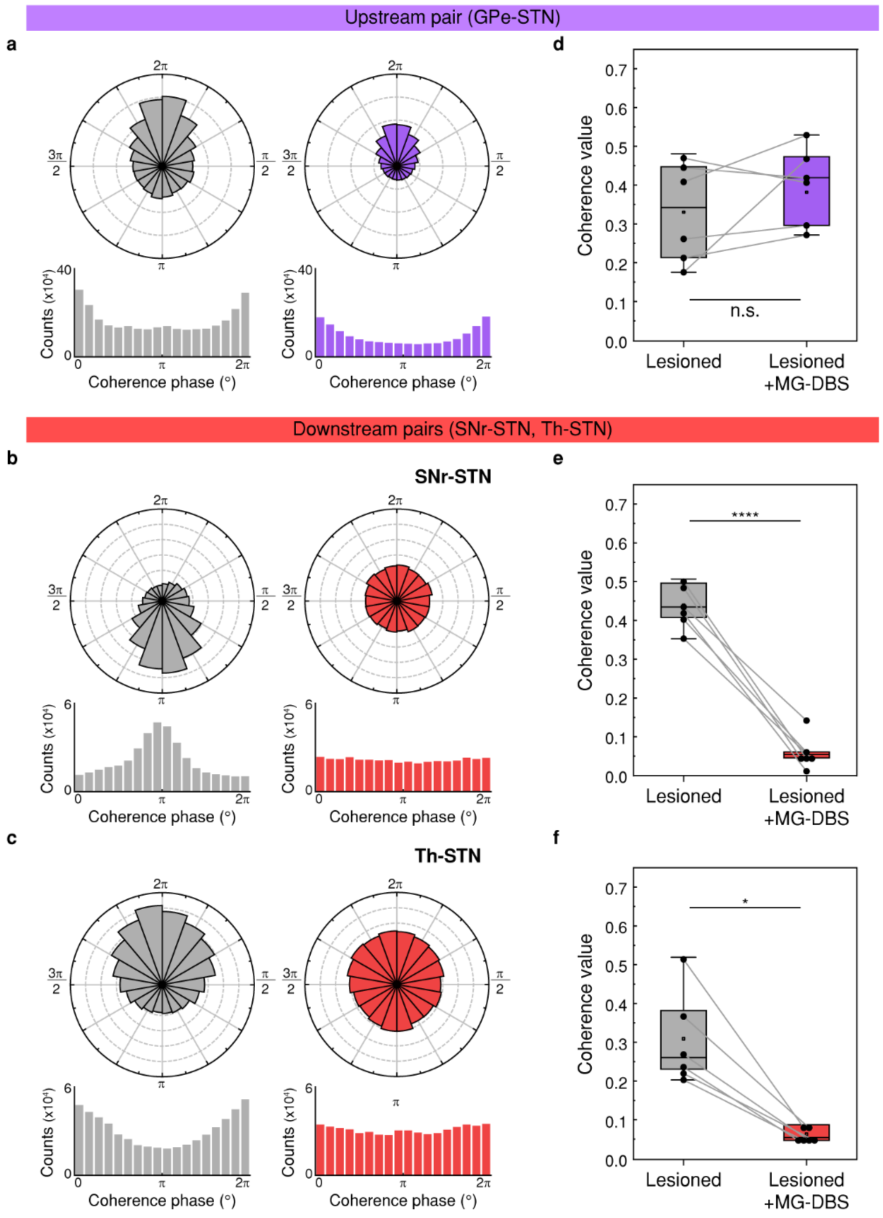

Figure S25. Selective disruption of pathological downstream coherence in the CBT circuit by MG-DBS. **a-c**, Representative histogram of coherent phase distributions for beta band oscillatory synchrony between region pairs: upstream GPe-STN (lesioned, r = 0.1857, θ = 3.640°; lesioned + MG-DBS, r = 0.2710, θ = 6.158°) (**a**), downstream SNr-STN (lesioned, r = 0.3505, θ = 168.3°; lesioned + MG-DBS, r = 0.0359, θ = 22.67°) (**b**), and downstream Th-STN (lesioned, r = 0.2430, θ = 1.048°; lesioned + MG-DBS, r = 0.0487, θ = 346.4°) (**c**) for lesioned

(grey) and lesioned + MG-DBS (purple, red) mouse. **d-f**, Quantification of coherence values for each pair: no significant change in upstream GPe-STN coherence after MG-DBS (d); significant reduction in SNr-STN (p = 0.0000071) (e) and Th-STN (p = 0.002) (f) coherence. Individual data points are overlaid (n = 6 mice). Box: 25th and 75th percentiles. Line: median. Whiskers: 1.5x interquartile range from the quartiles. Statistical differences were determined with paired t-test; *$p < 0.05$, ****$p < 0.0001$.

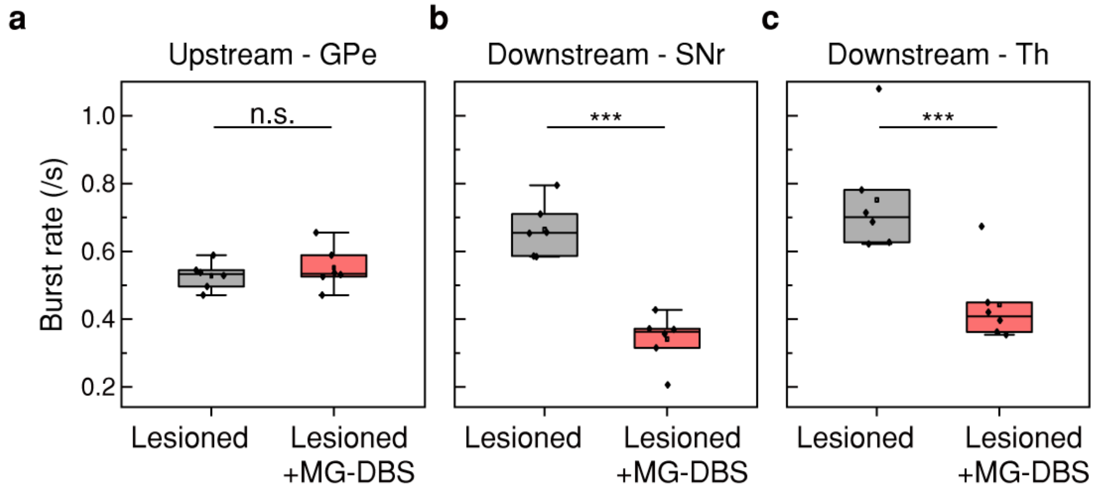

Figure S26. Region-specific modulation of burst rate by MG-DBS. **a-c**, Comparison of burst rate in upstream (GPe) (**a**) and downstream regions (SNr, $p = 0.0001$; Th, $p = 0.00015$) (**b, c**), while no significant change was observed in the upstream region. Individual data points are overlaid ($n = 6$ mice for each group). Box: 25th and 75th percentiles. Line: median. Whiskers: 1.5x interquartile range from the quartiles. Statistical differences were determined with paired t-test; ***$p < 0.001$.

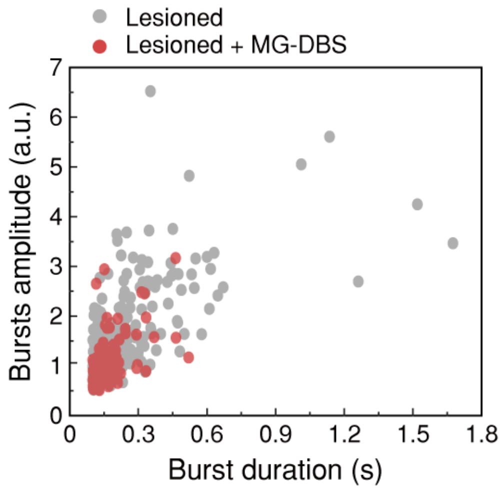

Figure S27. Representative scatter plots of detected beta burst in the STN. Each dot represents a single burst event, with amplitude plotted against burst duration. Grey and red dots indicate bursts recorded in the lesioned and lesioned + MG-DBS conditions, respectively.

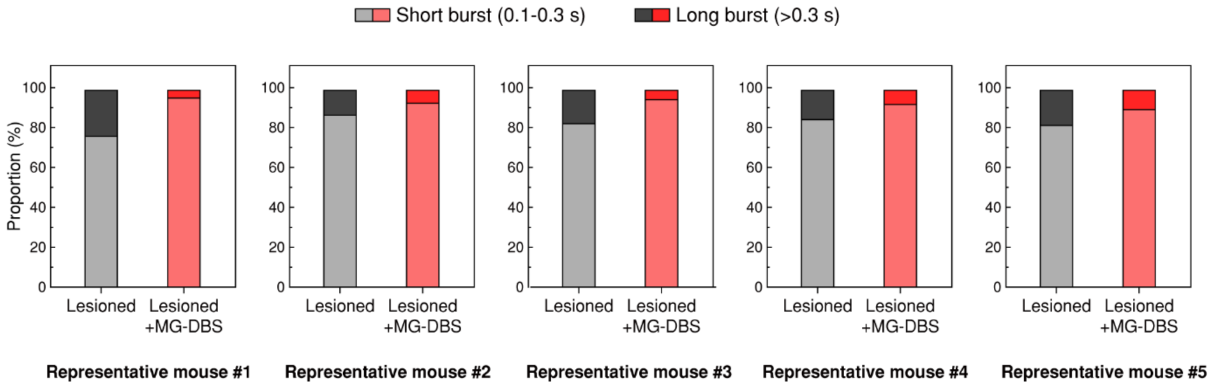

Figure S28. Overall distribution of short (0.1 - 0.3 s) and long (> 0.3 s) beta bursts in five representative mice. For each mouse, the proportion of short and long bursts is shown for the lesioned state and following MG-DBS treatment.

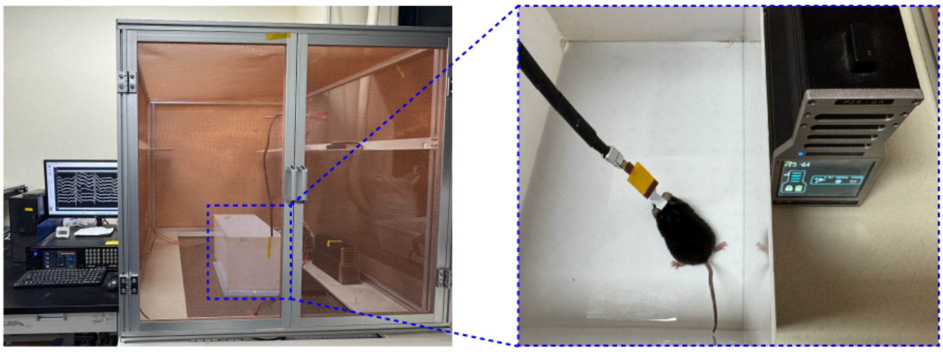

Figure S29. Photograph of the freely moving electrophysiological recording setup. Faradaic cage and external acquisition system (Left). Magnified view of a mouse connected to the implanted soft LM-based neural interface via a flexible flat cable during freely moving electrophysiological recording.

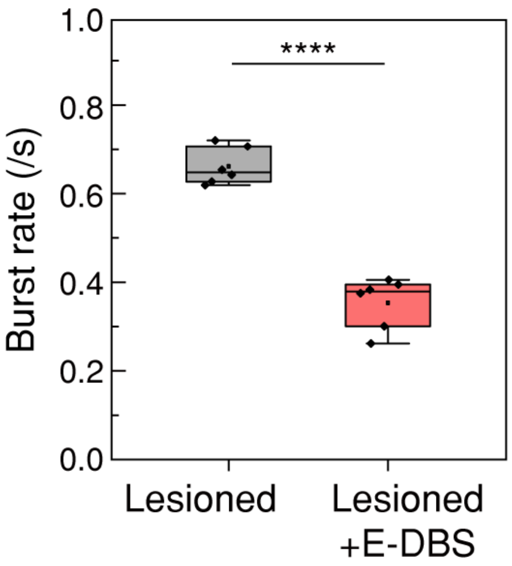

Figure S30. Change in burst rate in STN induced by E-DBS. (p = 3.58×10^{-6}) Individual data points are overlaid (n = 6 mice for each group). Box: 25th and 75th percentiles. Line: median. Whiskers: 1.5x interquartile range from the quartiles. Statistical differences were determined with paired t-test; ****$p < 0.0001$.

Captions for Movie S1

Movie 1. Comparative analysis of motor behaviors following MG-DBS and E-DBS. Video clips showing open-field locomotor activity of 6-OHDA lesioned mice at 1-hour intervals after stimulation with either MG-DBS (left, red trajectory) or E-DBS (right, blue trajectory). Movement trajectories were tracked during a 5-minute open-field test at each time point, with 1-minute representative segments shown here. Videos are shown at 3x speed.

Supporting References

[1] Jensen, W., Hofmann, U. G. & Yoshida, K. Assessment of subdural insertion force of single-tine microelectrodes in rat cerebral cortex. in *Proceedings of the 25th Annual International Conference of the IEEE Engineering in Medicine and Biology Society (IEEE Cat. No.03CH37439)* vol. 3 2168-2171 Vol.3 (2003).

[2] Srikantharajah, K. *et al.* Minimally-invasive insertion strategy and in vivo evaluation of multi-shank flexible intracortical probes. *Sci Rep* **11**, 18920 (2021).

[3] Wester, B. A., Lee, R. H. & LaPlaca, M. C. Development and characterization of in vivo flexible electrodes compatible with large tissue displacements. *J. Neural Eng.* **6**, 024002 (2009).

[4] Wang, Z. *et al.* A shear-lag model for laminated beams with extreme modulus mismatch between layers. *Mechanics of Materials* **188**, 104844 (2024).

[5] Cointe, C. *et al.* Scalable batch fabrication of ultrathin flexible neural probes using a bioresorbable silk layer. *Microsyst Nanoeng* **8**, 1–11 (2022).

[6] Li, P.-Y., Givrad, T. K., Holschneider, D. P., Maarek, J.-M. I. & Meng, E. A Parylene MEMS Electrothermal Valve. *Journal of Microelectromechanical Systems* **18**, 1184–1197 (2009).

[7] Park, Y.-G. *et al.* Liquid Metal-Based Soft Electronics for Wearable Healthcare. *Advanced Healthcare Materials* **10**, 2002280 (2021).

[8] Chung, W. G. *et al.* Ga-Based Liquid Metals: Versatile and Biocompatible Solutions for Next-Generation Bioelectronics. *Advanced Functional Materials* **n/a**, 307990.

[9] Thielen, B. & Meng, E. A comparison of insertion methods for surgical placement of penetrating neural interfaces. *J. Neural Eng.* **18**, 041003 (2021).
